# Proteomic profiling reveals that *ESR1* mutations enhance cyclin-dependent kinase signaling

**DOI:** 10.1038/s41598-024-56412-8

**Published:** 2024-03-22

**Authors:** Tommaso De Marchi, Chun-Fui Lai, Georgia M. Simmons, Isabella Goldsbrough, Alison Harrod, Thai Lam, Lakjaya Buluwela, Sven Kjellström, Christian Brueffer, Lao H. Saal, Johan Malmström, Simak Ali, Emma Niméus

**Affiliations:** 1 Division of Surgery, Oncology, and Pathology, Department of Clinical Sciences, Lund University, Solvegatan 19, 22362 Lund, Sweden; 2 Department of Surgery and Cancer, Imperial College London, Hammersmith Hospital Campus, Du Cane Road, London, W12 0NN UK; 3 Department of Biochemistry and Structural Biology, Center for Molecular Protein Science, Lund University, Solvegatan 19, 22362 Lund, Sweden; 4 Swedish National Infrastructure for Biological Mass Spectrometry – BioMS, Lund, Sweden; 5 Division of Oncology, Department of Clinical Sciences Lund, Lund University, Medicon Village, 22381 Lund, Sweden; 6 Division of Infection Medicine, Department of Clinical Sciences Lund, Faculty of Medicine, Lund University, Klinikgatan 32, 22184 Lund, Sweden; 7 Department of Surgery, Skåne University Hospital, Lund, Sweden

**Keywords:** Hormones, Proteomics, Cancer

## Abstract

Three quarters of all breast cancers express the estrogen receptor (ER, *ESR1* gene), which promotes tumor growth and constitutes a direct target for endocrine therapies. *ESR1* mutations have been implicated in therapy resistance in metastatic breast cancer, in particular to aromatase inhibitors. *ESR1* mutations promote constitutive ER activity and affect other signaling pathways, allowing cancer cells to proliferate by employing mechanisms within and without direct regulation by the ER. Although subjected to extensive genetic and transcriptomic analyses, understanding of protein alterations remains poorly investigated. Towards this, we employed an integrated mass spectrometry based proteomic approach to profile the protein and phosphoprotein differences in breast cancer cell lines expressing the frequent Y537N and Y537S ER mutations. Global proteome analysis revealed enrichment of mitotic and immune signaling pathways in ER mutant cells, while phosphoprotein analysis evidenced enriched activity of proliferation associated kinases, in particular CDKs and mTOR. Integration of protein expression and phosphorylation data revealed pathway-dependent discrepancies (motility vs proliferation) that were observed at varying degrees across mutant and *wt* ER cells. Additionally, protein expression and phosphorylation patterns, while under different regulation, still recapitulated the estrogen-independent phenotype of ER mutant cells. Our study is the first proteome-centric characterization of *ESR1* mutant models, out of which we confirm estrogen independence of ER mutants and reveal the enrichment of immune signaling pathways at the proteomic level.

## Introduction

Most breast cancers express the estrogen receptor (ER; *ESR1* gene), a steroid hormone receptor that acts as a transcription factor once bound by estrogens. ER positive breast cancers are treated with endocrine agents, which either block or degrade the receptor, or reduce systemic estrogen levels (aromatase inhibitors, AI)^1,2^.

About 30% of patients with ER-positive breast cancer treated with endocrine therapies develop recurrent disease, which is often refractory to further endocrine treatments. Several mechanisms of endocrine therapy resistance have been identified, such as alterations in expression of ER co-activators (e.g. AIB1/SRC-3)^3^, augmentation of other signaling pathways (e.g. ERK/MAPK)^4^, or the expression of mutant ERs^5–7^.

*ESR1* mutations have been reported in 11–55% of metastatic ER-positive breast cancers, mostly within the ligand-binding domain of the receptor (e.g. Y537N/C/S and D538G), and promote constitutive and ligand-independent activation of the ER^8^. On top of this, *ESR1* mutations have been shown to activate other signaling pathways (e.g. interferon response)^5,9–11^, suggesting additional transcriptional effects when compared to the *wt* receptor (reviewed in^12,13^). The genetic and transcriptomic alterations associated with expression of ER mutants have been widely investigated in breast cancer cell lines in which ER mutants arise spontaneously^14^, following ectopic expression^5,6,8^ or by CRISPR-Cas9-directed knock-in mutagenesis of the endogenous *ESR1* gene^9,10,15,16^. These studies have shown that *ESR1* mutants often upregulate proliferation-related genes (e.g. cyclins), immunomodulatory signaling factors (e.g. Type I interferon), and display altered patterns of chromatin accessibility that affect transcriptional programs of other transcription factors other than the ER (e.g. OCT1 and CTCF)^10,15^. Despite this, profiling of protein abundance and phosphorylation levels have not been explored in *ESR1* mutant breast cancers.

**Figure 1:**
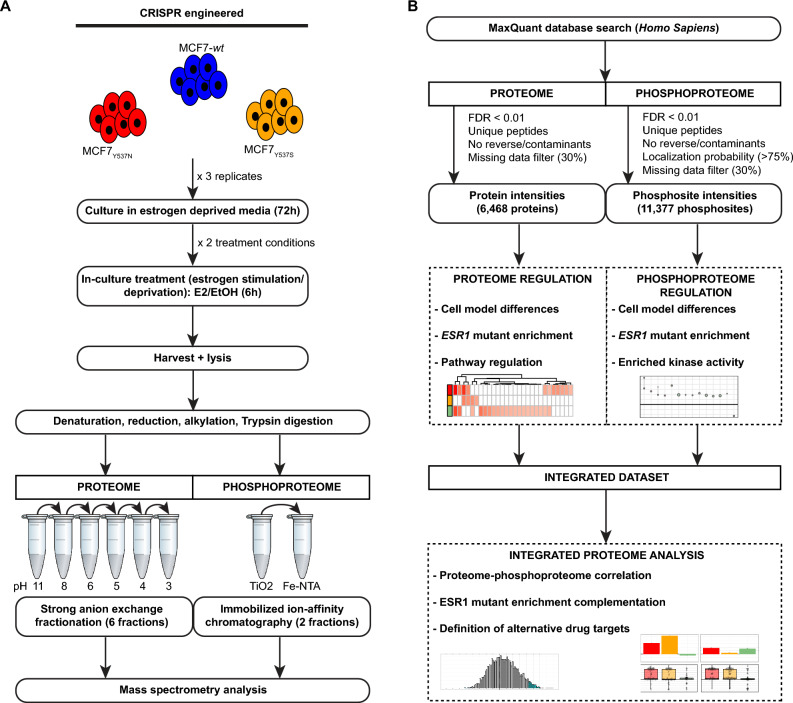
Experimental and data analysis workflow. We employed cell models expressing *ESR1* mutations (MCF7_Y537N_, MCF7_Y537S_) and their isogenic *wt* ER counterparts. Cells were cultured in estrogen-free media for 72 h, then treated with estradiol (E2) or vehicle (EtOH) for 6 h before harvesting. Cells were lysed, protein extracts digested, fractionated, and analyzed by MS. Proteome and phosphoproteome data was searched with MaxQuant, resulting output was filtered (decoy, false identification, missing data), and employed for downstream comparative analyses. Datasets were subsequently integrated to define, based on overlapping enrichment, alternative drug targets for validation. Acronyms: E2: 17-β-estradiol; ER: estrogen receptor; EtOH: ethanol; MS: mass spectrometry.

Mass spectrometry (MS)-based proteomic technologies have allowed in-depth characterization of cancer and other diseases^17,18^, and through integrated approaches such as proteogenomics, enable improved definition of tumor subgroups and the elucidation of altered pathways and driver mutations that could define cell fate and patient outcome^19,20^. Moreover, protein-centered analyses allow direct interrogation of the effectors of biological systems, facilitating the identification of biomarkers or drug targets^21,22^. On top of this, the analysis of phosphorylated proteins (phosphoproteomics) is capable of defining protein activation dynamics that modify the functional properties of cells (e.g. phosphorylation patterns during mitotic states)^23,24^. With these points in mind, we employed a combined proteomic and phosphoproteomic approach to define protein and phosphorylation patterns in *ESR1* mutant models under estrogen deprivation (mimicking AI treatment) and estrogen stimulation conditions. By linking gene/protein expression changes to the activity of specific kinase signaling cascades might identify alternative therapeutic targets. We used MCF7 cells in which CRISPR-Cas9 methodology was used to introduce the Y537S^9^ and the Y537N^25^ mutations into the endogenous *ESR1* gene (Fig. 1). In addition to this, we explored the proteome dynamics in a recently described T47D model bearing the Y537S mutation^10,15^. We show that the estrogen independent phenotypes of these models have different repercussions for proteome and phosphoproteome dynamics, depending on the cell model and type of mutation, though still promoting proliferation through inter-connected pathways.

## Results

### ESR1 mutation analysis and external dataset comparison

**Figure 2:**
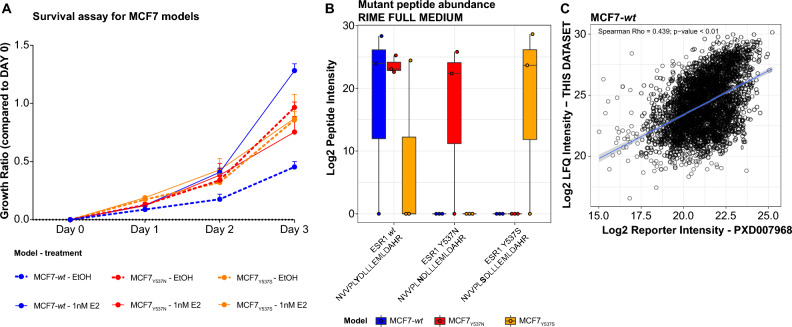
Mutational analysis and comparison proteomic profiles in this study to public datasets. Panel A represents results of Sulforhodamine-B growth assays of MCF7 cells. Cells, pre-cultured in estrogen-free medium, were treated with estrogen (E2; 1 nM) or an equal volume of vehicle (veh; ethanol). Panel B displays MS-based detection of mutant (Y537N: NVVPL**N**DLLEMLDAHR; Y537S: NVVPL**S**DLLEMLDAHR) and *wt* ER (NVVPL**Y**DLLEMLDAHR) peptides in MCF7 models, performed using MS analysis of ER immune-precipitates (RIME MS). Panel D depicts scatter plots of overall protein abundance correlations between the proteomic data generated in this study and a previously published datasets and MCF7-*wt* (PXD007968). Acronyms: E2: 17-β-estradiol; ER: estrogen receptor; EtOH: ethanol; MS: mass spectrometry; RIME: rapid immunoprecipitation of endogenous proteins.

In this study, we employed breast cancer cells expressing commonly observed *ESR1* mutations (Y537N and Y537S) and *wt* ER counterparts and evaluated their growth in the presence/absence of estrogen (E2; Fig. 1). MCF7_Y537N_ (ref^25^) and MCF7_Y537S_ (ref^9^) cells, generated using CRISPR-Cas9 approaches, were estrogen-independent for growth (Fig. 2A). Genomic analysis and RT-qPCR have demonstrated that the MCF7_Y537N_ and MCF7_Y537S_ clones are heterozygous for their mutation^9,25^. MS analysis of cell lysates after rapid immunoprecipitation of endogenous proteins (RIME) using an ER antibody confirmed the presence of the relevant mutant and *wt* ER peptides in the MCF7_Y537N_ and MCF7_Y537S_ clones (Fig. 2B). Mutant peptides were absent in MCF7-*wt* cells. Of note, we did not detect any new ER interacting partner in *ESR1* mutant over *wt* cells.

Next, we set to define estrogen dependent and independent changes by global proteome and phosphoproteome analysis. For this, we cultured MCF7 models in estrogen-free medium for 72 h (Fig. 1) prior to the addition of E2 or vehicle (ethanol, EtOH). Treatment duration was set to 6 h, consistent with the maximal activity/stability of the ER transcriptional complex^22^. This analysis generated a list of 6468 proteins (Table S1) and 11,746 phosphopeptides (Table S2), respectively.

Given that potential discordance between proteomic datasets can arise from use of different cell batches, non-identical culture conditions, sample preparation (e.g. protein labelling *vs* label-free) and MS methodologies (e.g. instrument type, gradient length), we compared our proteomic data with those reported previously. The proteome profiles obtained for our MCF7-*wt* cells correlated well with the previously reported MCF7 proteome (Fig. 2C)^22^. The agreement between our protein expression profiles and those reported previously provided confidence for taking forward our datasets for further analysis.

As an additional dataset, we analyzed our MCF7 Y537S models in parallel with previously described T47D Y537S mutant^10,15^. Here, cells were stimulated with E2 or vehicle (dimethyl sulfoxide, DMSO) for 6 h and 24 h so to better capture early and late estrogen effects. As a baseline, proteomic data of these cells grown in full medium was also acquired. This second dataset is reported in Tables S3–4.

### Protein abundance and phosphorylation differences reflect the ESR1 mutational status and estrogen treatment

**Figure 3:**
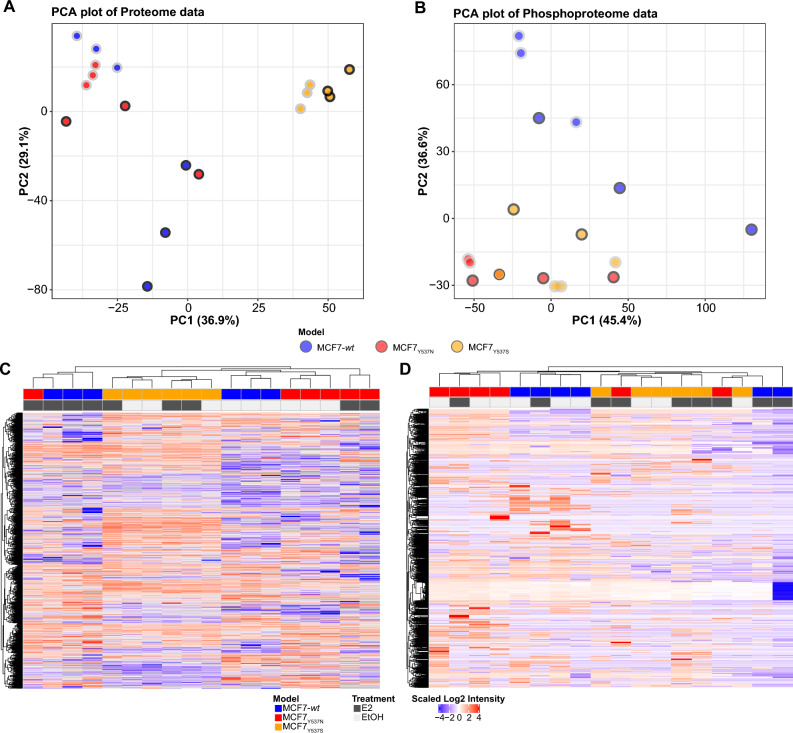
Principal Component analyses of proteome and phosphoproteome data. Figure displays global PCA analyses for proteome (panel A) and phosphoproteome (panel B) data for all cell models under estrogen stimulation (E2) and deprivation (EtOH) conditions. Panels C and D display hierarchical clustering heatmaps based on proteome and phosphoproteome data, respectively. Acronyms: E2: 17-β-estradiol; ER: estrogen receptor; EtOH: ethanol; MS: mass spectrometry.

We determined whether estrogen treatment and/or mutational status alter global protein and protein phosphorylation levels. PCA analysis of proteome data showed clear segregation of MCF7 *wt* cells dependent on estrogen treatment, while a weaker effect of estrogen was observed for MCF7_Y537N_ cells (Fig. 3A). By contrast, the PCA analysis indicated considerable change in the MCF7_Y537S_ proteome when compared with MCF7-*wt*, with little or no difference based on estrogen stimulation. PCA analysis of the phosphoproteome more clearly distinguished MCF7-*wt* from its mutants. Here MCF7-*wt* displayed differential clustering based on E2 treatment. MCF_Y537N/Y537S_ co-clustered away from their *wt* counterpart, indicating smaller changes in phosphorylation that are dependent on estrogen, but also a significant shift in phosphorylation patterns when compared to *wt* cells, suggesting alterations in different kinase signaling cascades independent of estrogen (Fig. 3B). These groupings were confirmed by hierarchical clustering analyses (Fig. 3C,D).

When assessing these differences in our second dataset, we observed distinct clustering between MCF7 and T47D cells, with no co-clustering based on *ESR1* genotype (Fig. S1A). PCA analysis of treated cells showed little distance in the PCA plane between cells harvested at 6 h, though vehicle and estrogen treated cells still formed sub-clusters. At 24 h cluster separation was more evident for both *wt* and mutant cells and was consistent for both MCF7 and T47D models (Fig. S1B–C). Phosphoproteome data of this second set displayed the same clustering, with clear separation between T47D and MCF7 models in the full medium subset, as well as separation between E2 and vehicle treatments across the two time points (Fig. S1D–F).

Collectively, our data suggest that proteome and phosphoproteome alterations depend not only on the expression of *wt* or mutated forms of the ER and its activation by the presence of estrogens, but also on cell model. On this, metascape analysis^26^ of ANOVA significant proteins and phosphosites (*p* < 0.05) between MCF7 and T47D cells (full medium subset) showed enrichment in proteins involved in transcription and RNA regulation both at the proteome and phosphoproteome levels (Fig. S2A–B). In addition to this, while differences between estrogen and vehicle stimulated cells are observed at the 6 h time point, these become more evident at 24 h, where late estrogen effects likely take place.

**Figure 4:**
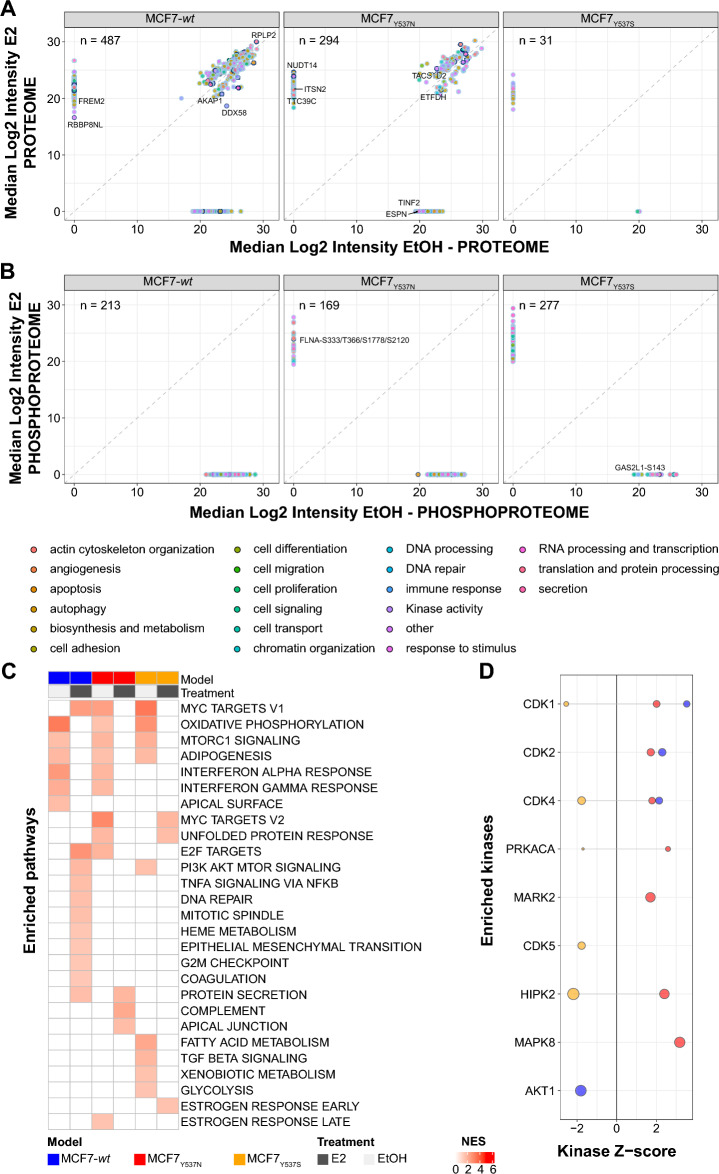
Protein and phosphosites-centered analysis of response to estrogens. Figure displays proteome and phosphoproteome abundance shifts across *wt* and mutated ER cell models after treatment with EtOH and E2. Panels A and B display scatter plots of differentially expressed (t-test adjusted *p-value* < 0.05) proteins and phosphosites between E2 and EtOH conditions per each cell model. Dark blue contoured dots represent *ESR1* target genes (source: ENCODE), while filled dots represent pathway annotation. Panel C show heatmap of GSEA results for MCF7 models. Panel D shows KSEA results for MCF7 models where phosphorylation patterns were compared between E2 and EtOH conditions. Positive KSEA Z-scores define enrichment under estrogen, while negative scores portray EtOH enrichment. Acronyms: E2: 17-β-estradiol; ER: estrogen receptor; EtOH: ethanol; MS: mass spectrometry; GSEA: gene set enrichment analysis; KSEA: kinase substrate enrichment analysis.

In order to further confirm the different response to estrogens across cell models we performed differential expression analysis on our proteome and phosphoproteome data, which showed that all cell models displayed changes in protein expression (t-test adjusted *p-value* < 0.05) between E2 and vehicle conditions (Table S5), with the exception of MCF7_Y537S_, in line with PCA results (Fig. 4A). In the phosphoproteome dataset, differentially expressed phosphosites generally encompassed gains and losses (i.e. fully observed in one condition and completely absent in the other; Fig. 4B and Table S6), such as ARID3A phosphorylation at Ser77 observed in MCF7_Y537S_ (*p-value* < 0.01). These differences might be related to the different type of response to estrogen, and the consequent alteration of transcriptional programs, enacted across different cell models. On top of this, cells bearing different *ESR1* mutations have also shown variability in gene expression in relation to the amino acid type and position (e.g. D538G and Y537S)^15,16^, which can then explain these dissimilarities.

Next, we performed Gene Set and Kinase-Substrate Enrichment Analysis (GSEA^27^, KSEA^28^) on our proteome and phosphoproteome data, respectively (Tables S7–8). Here, cell proliferation (e.g. mTORC1 signaling, MYC targets) and metabolism (e.g. oxidative phosphorylation) were enriched in mutant models in estrogen deprivation conditions, while in *wt* cells these gene sets were upregulated after E2 stimulation. Analysis for Reactome terms displayed similar results, with numerous pathways significantly enriched after estrogen in *wt* cells (Fig. S3A–B). Enrichment of cell cycle, transcription and signaling pathways were observed in *wt* cells under estrogen stimulation, while *ESR1* mutants showed the same enrichments under estrogen deprivation (Fig. S3C, Table S9). In this set, KSEA results revealed activation of CDKs (i.e. CDK1, CDK2, CDK4) after estrogen stimulation in MCF7 models, with the exception of MCF7_Y537S_, which displayed CDK upregulation under estrogen deprivation (Fig. 4C).

GSEA of our second dataset confirmed enrichment of ER signaling and proliferation pathways in both MCF7 and T47D *wt* models, with a more pronounced response in T47D cells. On top of this, while MCF7_Y537S_ treated with vehicle showed enrichment of pathways typically enriched in the wt counterpart when treated with estrogens, T47D cells showed a major response to estrogen stimulation, which stimulated immune (e.g. allograft rejection), proliferation (e.g. mTORC1 signaling), and metabolism (e.g. glycolysis) pathways (Fig. S4A). KSEA analysis of this additional set showed CDK activation after estrogens in MCF7-*wt* (CDK1/2/7), while the Y537S mutant showed positive but non-significant enrichment (Fig. S4B).

These results confirm that *ESR1* mutant cells operate through similar molecular pathways (e.g. proliferation) as their *wt* counterparts, especially after estrogen withdrawal.

### Cell proliferation and immune signaling pathways are enhanced in ESR1 mutant cells

**Figure 5:**
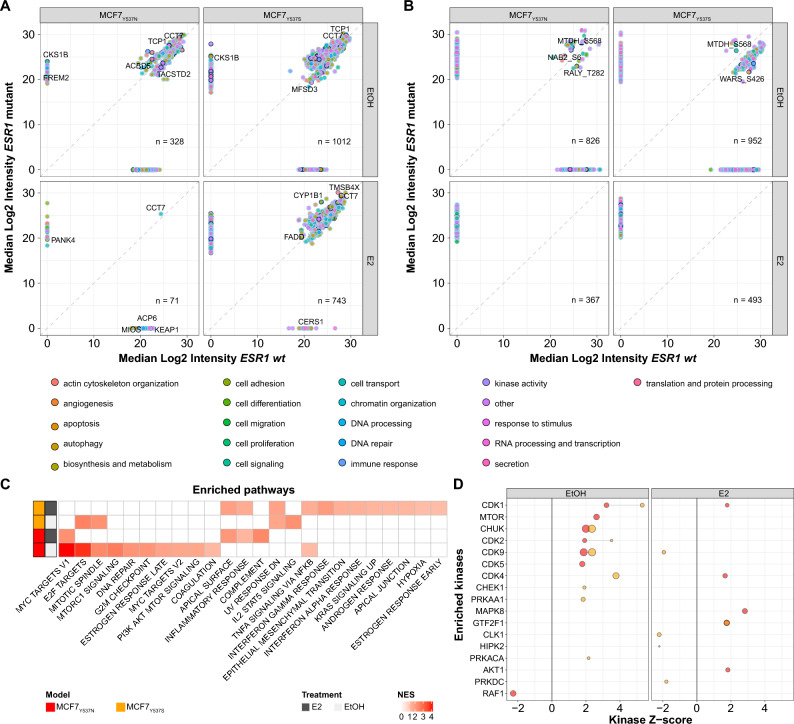
Protein and phosphosite enrichment analysis of *ESR1* mutants. Protein and phosphosite enrichment analyses were performed on *ESR1* mutants in comparison with isogenic *wt* ER cells across E2 and EtOH conditions. Panels A and B display scatter plots of differentially expressed (t-test adjusted *p-value* < 0.05; dark contour dots: ER targets; filled dots: pathway annotation) protein and phosphosites between mutant and *wt* cells after EtOH (left) and E2 (right). Panel C show heatmap of GSEA analyses MCF7 mutants. Panel E represents KSEA analyses results (mutant *vs wt*) after EtOH (left) and E2 (right) treatment. Positive KSEA Z-scores score define enrichment in *ESR1* mutants, while negative scores portray enrichment in wt cells. Acronyms: E2: 17-β-estradiol; ER: estrogen receptor; EtOH: ethanol; MS: mass spectrometry; GSEA: gene set enrichment analysis; KSEA: kinase substrate enrichment analysis.

Having established that cell models expressing *ESR1* mutations display an altered response to E2, we investigated specific differences between each mutant and its *wt* ER isogenic counterpart (Fig. 5A,B and Tables S10–11). Here, commonly dysregulated proteins under estrogen deprivation across mutant cells mapped to pathways related to protein stabilization and folding (e.g. CCT7) as well as telomerase activity related networks (e.g. TCP1; source: DAVID v2021q1^29^; Fig. 5A). Additionally, several *ESR1* targets (e.g. FREM2, CKS1B) were enriched in ER mutants, especially at the protein level under estrogen deprivation, suggesting ligand-independent activation of the ER pathway in these models. These results show that cells expressing ER mutants not only display dysregulation of *ESR1* signaling, but also show changes in other cellular pathways^5,6^. Closer investigation of genes responsive to ER (MSigDB v2021.1 ; ref^30^) confirmed not only a different regulation for each mutant over *wt* cells, but also showed differences between protein and phosphosite abundances (Fig. S5), with little change at the protein level but marked differences within the phosphoproteome.

GSEA analyses performed on proteome data pointed out that differentially expressed proteins in *ESR1* mutants related to cell cycle, proliferation, and immune signaling pathways (Fig. 5C). Similar results were observed when interrogating the Reactome database, where mutants showed pathway enrichment over *wt* cells mostly under estrogen deprivation (Fig. S6 and Table S12). Here, cell cycle and TGFβ pathways were found significantly enriched in mutants in absence of estrogens (Fig. S6B), while E2 stimulation highly enriched these networks in *wt* cells (Fig. S6C). Enrichment of immune signaling pathways, such as the Interferon-related signaling network, were described in previous studies and linked to immunomodulation of the cancer microenvironment^10^. Closer investigation of the reported gene sets revealed immune signaling protein enrichment in ER mutants (e.g. IFITM1; Fig. S7A–B). These gene sets displayed a large overlap with our GSEA results, especially with the INTERFERON GAMMA RESPONSE gene set, which displayed a significant enrichment in the MCF7_Y537S_ model (Fig. S7C).

Our second dataset showed similar results, with proliferation promoting pathways (e.g. MYC targets, E2F signaling) enriched in *ESR1* mutants at 6 h under estrogen deprivation. Additionally, a higher number of enriched pathways was found in T47D models when compared to MCF7 cells. Despite of this, both models showed similar enrichment trends, with pathways enriched in mutant cells under estrogen deprivation found significant in *wt* cells under estrogens (e.g. MYC targets, interferon gamma response, PI3K/Akt/mTOR signaling; Fig. S8A). Further investigation of *ESR1* mutant in a clinical breast cancer cohort (SCAN-B^31^; n cases = 27; n controls = 108, see *Methods* for details) largely confirmed our cell model results (top enriched pathways: E2F targets, G2M checkpoint, MYC targets, and mTORC1 signaling; Fig. S8B).

KSEA analyses on the phosphoproteome dataset comparing ER mutants and their *wt* isogenic cells revealed multiple kinase activations, with CDK1/2/4/5/9, and mTOR enriched in MCF7 mutant models under estrogen deprivation. Moderate enrichments of CDKs and other kinases (e.g. MAPK8) over *wt* cells were observed under estrogen stimulation (Fig. 5D). Closer inspection of CDK network expression showed a higher abundance of CDK4 and CDK6 in MCF7_Y537S_ at the protein level and regardless of treatment (Fig. S9A), while phosphorylation of CDKs was evident in this model under estrogen deprivation (Fig. S9B). CDK target enrichment was especially pronounced in mutant cells under estrogen deprivation (Fig. S9C), with abundance enrichments at the phosphosite level rather than the protein one (Fig. S9D–E). We assumed these enrichments might relate to Cyclin protein expression, which showed increased expression in MCF7_Y537S_ at the protein level (e.g. CCNY, CCND1; Fig. S9F), but only wt cells showed increased phosphorylations (under estrogens; Fig. S9G). Our additional dataset confirmed CDK enrichment in *ESR1* mutants over *wt* cells, both in full medium and after estrogen or vehicle treatment (e.g. CDK2, CDK4; Fig. S10).

Altogether these data confirm that ER mutants appear to enact their phenotype through the upregulation of proliferation and mitotic pathways, which in turn are responsible for growth in estrogen deprivation conditions. Despite these results, inhibition of key pro-mitotic pathways (i.e. mTOR and CDK inhibition) did not show significant differences in growth inhibition effects comparing *ESR1* mutant cells to *wt* MCF7 (Fig. S11), leading us to believe other factors might be involved in sustaining proliferative signaling and promoting resistance to mTOR and CDK inhibitors^32–34^. To obtain an additional angle to define these putative factors, we wondered whether integration of the proteomic data layers might provide additional information for target selection.

### Integrated analysis shows model-dependent proteome-phosphoproteome regulation of splicing machinery

Having assessed that the proteome and phosphoproteome undergo different levels of regulation between *ESR1* mutant cells but converge at the pathway level, we investigated our merged proteome and phosphoproteome data. The resulting dataset was particularly enriched in RNA processing proteins (Fig. S12A). Spearman correlations over the whole dataset revealed that protein and phosphosite abundance were either de-coupled (no correlation) or positively correlated (Fig. S12B and Table S13). Enrichment analysis for Gene Ontology Biological Process (GOBP) terms showed that RNApol-II transcription pathways displayed positive correlations, while cell cycle and splicing attributes showed decoupled or negative correlation (Fig. S12C). Distributions of Spearman coefficients displayed different trends in each model, where MCF7-*wt* and MCF7_Y537N_ models showed negatively oriented correlation distributions, while in the MCF7_Y537S_ mutant the distribution was centered on 0 (Fig. S12D). Here, GOBP enrichment analyses displayed similar results to the one performed on the whole dataset (Fig. S12E). Agreement between proteome and phosphoproteome appeared to be model-dependent, with no discernible impact based on treatment.

This data shows that while significant correlations between proteome and phosphoproteome changes are mostly positive, cells expressing *ESR1* mutations often display shifts in protein-phosphorylation regulation when compared to their *wt* counterparts, supporting the idea that kinase signaling plays an important role in regulating protein abundance.

### Proteomic and phosphoproteomic changes pinpoint targets for alternative therapies

**Figure 6:**
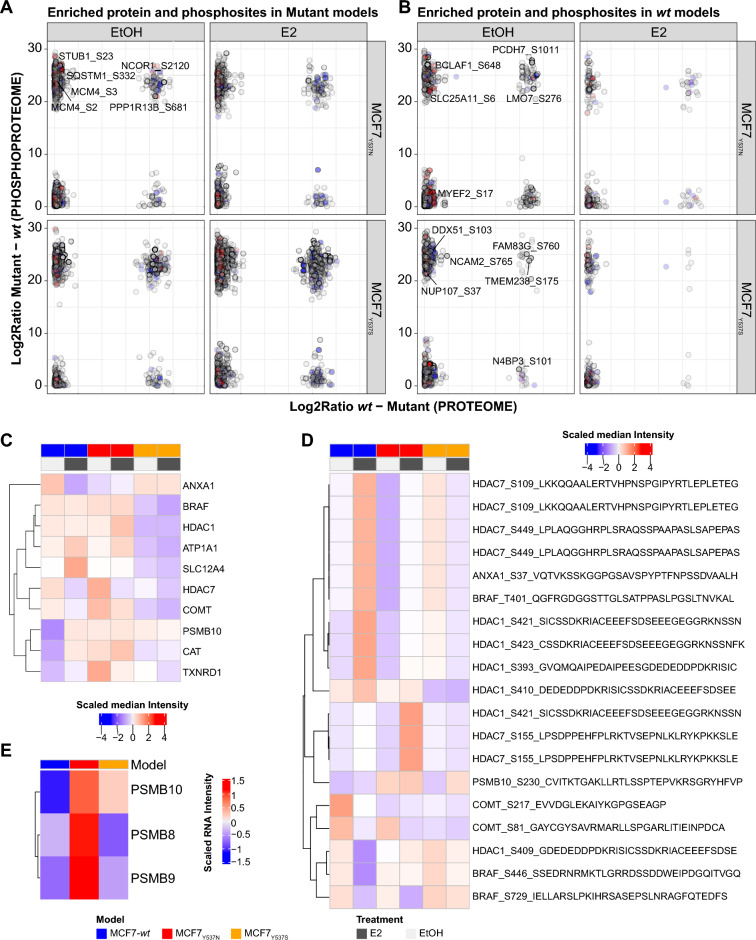
Integrated analysis of ER mutant cells. Figure displays the results of the combined analysis of mutant enrichments across all data layers. Panels A and B show enrichments of protein and phosphosites in ER mutants and *wt* ER isogenic cells (significance in both data layers is represented in full color; red dots: ER interactors; blue dots: ER targets). Panels C and D represent expression heatmaps of essential gene products targetable by FDA approved drugs at the proteome and phosphoproteome level, respectively. Panel E displays transcript enrichment of the immunoproteasome in ER mutants out of RNA-sequencing data. Acronyms: E2: 17-β-estradiol; ER: estrogen receptor; EtOH: ethanol; MS: mass spectrometry; GSEA: gene set enrichment analysis; KSEA: kinase substrate enrichment analysis.

**Figure 7:**
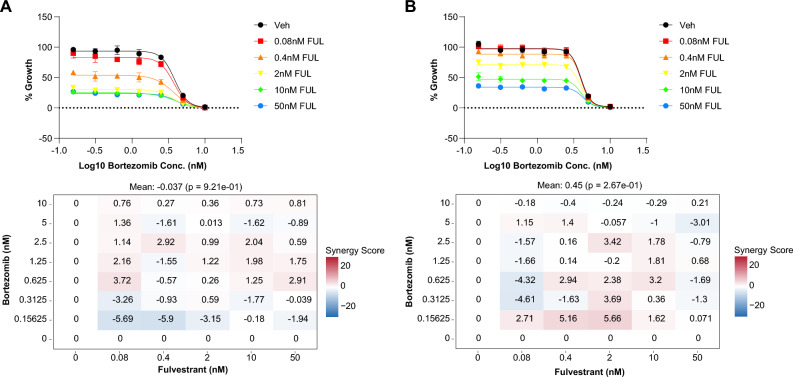
Drug synergy assessment with endocrine therapy. Figure displays the response curves for proteasome inhibitor Bortezomib in combination with Fulvestrant (upper plots) for MCF7-*wt* (panel A) and the MCF7_Y537S_ (panel B) models. Synergy scores were then calculated based on the Bliss model (see *Methods* section for details) and displayed for every concentration in heatmaps (lower plots).

Having established that proteome and phosphoproteome of *ESR1* mutant cells display a degree of co-regulation when compared to *wt* models, we wondered whether the overlap between protein and phosphosite enrichments might pinpoint alternative targets for therapy. Here, we observed that subsets of proteins were enriched at both the proteome and phosphoproteome levels in our ER mutant (e.g. NCOR1), with dysregulation of both ER responsive genes and interactors (e.g. RBBP7, ADNP; Fig. 6A,B). We argued that selection of alternative therapeutic targets should include not only our datasets, but also functional assays. With this in mind, we cross-referenced our proteome and phosphoproteome datasets against CRISPR screen-derived ER mutant essential genes^11^ for which FDA approved drugs are available (source: www.proteinatlas.org/humanproteome/druggable)^35^. In this subset, protein and phosphosite data across *wt* and mutant ER models showed enrichment of proteins involved in gene expression regulation (e.g. HDAC1/7), metabolism (e.g. CAT), and cytoskeletal (e.g. MAP4) proteins. Of note, proteome data pinpointed PSMB10 as constitutively enriched in *ESR1* mutants, regardless of treatment (Fig. 6C), while protein phosphorylation showed upregulation of this protein especially in ER mutant MCF7 cells (Fig. 6D). PSMB8, PSMB9 and PSMB10 are part of the immunoproteasome complex, which is activated in response to interferon gamma signaling, and has been previously associated to cancer cell survival and progression^36–39^. Having observed enrichment of the immunoproteasome complex and immune signaling pathways in our proteomic data, as well as transcript upregulation in ER mutant MCF7 models out of RNA-sequencing (RNA-Seq) data (Fig. 6E)^9^, we determined whether inhibition of the immunoproteasome could constitute an alternative therapeutic strategy. We employed commercial inhibitors of the proteasome, which act by blocking (reversibly or irreversibly) proteasome activity, promoting the accumulation of ubiquitinated proteins and inducing cell cycle arrest and apoptosis. The observed effect on cell growth for the three commercial inhibitors (i.e. Bortezomib, Carfilzomib, Ixazomib) did not show any difference between cells expressing *wt* and mutated *ESR1* as single agent treatment (Fig. S13A–C). When testing Bortezomib combination treatment with Fulvestrant, as also tested in phase II clinical trials^40^, we observed an additive effect between the two drugs, though inhibition effects were much more pronounced in *wt* cells when compared to the MCF7_Y537S_ model (Fig. 7). This might be due to the lack of specificity of these inhibitors in targeting the immunoproteasome (i.e. PSMB8/9/10). Alternatively, the impact of these upregulated proteins in elevated interferon signaling would facilitate metastatic processes by remodeling tumor-associated immune responses in vivo, as has been proposed^10^ rather than being critical for enhanced proliferation. On top of this, other in vitro factors dependent on 2D culture (reviewed in^41^) might be responsible for the limited response to the tested drugs.

## Discussion

About three quarters of breast cancer cases express the ER, which drives their growth and proliferation, hence impacting patient prognosis. While treatment of primary tumors remains effective through surgery and adjuvant endocrine therapies (in particular AIs), recurrent breast cancers remain the most frequent cause of death^1,42^. Recurrent tumors are often refractory to therapies due to acquired mutations, cross-talk mechanisms, etc.^2^, and new therapeutic strategies are needed. Mutations in the ligand binding domain of the ER have been shown to confer resistance to AI therapy by promoting ligand-independent activation of the receptor, but also by altering cellular signaling beyond the ER pathway^5,8^. So far, molecular changes enacted by *ESR1* mutations have been largely evaluated through epigenetic and genomic technologies^9,14^. Here we employed an integrated proteomic approach to characterize protein and phosphorylation networks in *ESR1* mutant cells across estrogen stimulation and deprivation conditions to extrapolate key mechanisms responsible for therapy resistance.

In this study, we employed cancer cell models engineered to harbor *ESR1* mutations (MCF7_Y537N/Y537S_), as well as an external set of mutated T47D (Y537S mutation). Each model was analyzed in relation to protein and phosphosite changes in response to estrogen deprivation and stimulation conditions, and against its isogenic counterpart expressing *wt* ER.

Our global analysis of the proteome and phosphoproteome datasets showed that mutant cells clustered separately not only from their *wt* counterparts. Here, MCF7 and T47D cells displayed variable responses to estrogen deprivation and stimulation in relation to differential protein expression, phosphorylation patterns, and pathway activation. Despite of this, common activation of oncogenic pathways (e.g. *MYC* response genes) was observed across our mutant models, complementing previous observations^10,15^. For this, while cell line-specific responses were not surprising, the integrative analysis of different cell models provides an outlook into cancer diversity and enables to discriminate proteins and pathways common to *ESR1* mutants.

When assessing response to estrogens, we noted that only MCF7_Y537S_ did not show any changes in terms of differentially expressed proteins, indicating this mutant as unresponsive to estrogen signaling. Conversely, the MCF7_Y537N_ mutant displayed estrogen response similar to its *wt* counterpart (Figs. 3 and 4), which could reflect the observed higher sensitivity to SERDs when compared to the MCF7_Y537S_ mutant^5,25^. Despite this, a marked enrichment of proliferation (e.g. *MYC* targets) and metabolism (e.g. oxidative phosphorylation) pathways was observed across MCF7 mutants. In addition, phosphoproteomic analysis confirmed the activation of CDKs across mutant models and in *wt* cells under estrogen stimulation, directly linking the growth phenotype of these cells and molecular changes at the protein level. Our second dataset comprising T47D_Y537S_ showed a higher response to estrogen stimulation, where we found enrichment of immune signaling and metabolism on top of proliferation pathways (Fig. S4A). The relationship between *ESR1* mutant expression and CDK activity has been pinpointed in previous studies, especially in relation to CDK7-dependent phosphorylation of Ser118, which has activating effects on the ER^9^. While ER phosphosites were not detected in our dataset, we nonetheless demonstrate a significant contribution of CDKs towards the establishment of the estrogen independent phenotype in *ESR1* mutant breast cancer cells.

Upon comparing mutant to *wt* cells, we observed distinct protein abundance changes, even among ER targets (e.g. CRTC1). Here, we observed moderate upregulation of ER targets in *ESR1* mutants over *wt* cells at the proteome level, but more evident changes were detected for phosphosites. On top of this, proliferation (e.g. E2F targets) and immune signaling (e.g. interferon) pathways were enriched in mutant cells, with kinase activity analyses pointing out a major role of CDKs and mTOR in driving the increased phosphorylation patterns. These findings were confirmed not only in our analysis of the T47D_Y537S_ model but also in a set of primary breast tumors, where proliferation pathways were highly enriched in mutant tumor cells (Fig. S8). Growth factor-related Tyr kinases and CDK activity have been previously linked to breast cancer endocrine resistance through altered phosphorylation patterns and modulation of ER signaling (reviewed in^7,43^). Recent studies observed increased activity of these pathways in *ESR1* mutants out of transcriptome analyses^9,14^. CDKs regulate cell cycle progression and activate (through phosphorylation) a series of proteins involved in RNA processing (e.g. splicing) and transcription (e.g. POLR2A). Inhibition of CDK4 and 6 currently constitutes the standard of care for recurrent ER positive breast cancers in combination with endocrine therapies, though progression under such treatment may develop through (e.g.) activation of Tyr kinase receptors^44^. No difference in sensitivity to CDK4/6 inhibition was observed between *wt* and *ESR1* mutant models, suggesting several proliferation axes operating in these models. While activation of these upstream kinases may vary, immune pathways in *ESR1* mutated tumors might have a prominent role in activating CDK signaling through cross-talk mechanisms^7,45^. Inflammation and immune signaling networks (e.g. Interferon-related pathways) have been linked to immunosuppressive macrophage infiltration in breast cancers with mutated ERs^10^. While immune signaling pathways may provide a selective advantage within the tumor microenvironment towards immune escape (e.g. Interferon gamma response), their activation may also be responsible for cell growth and proliferation via non-canonical activation of the mTOR pathway^45^, which enrichment has been repeatedly reported in *ESR1* mutants together with metabolic reprogramming and enhance cell growth and proliferation^9,14,33,34^.

Building on the idea that proliferation-associated pathways were found enriched at the protein abundance and phosphorylation levels, we overlaid the proteome and phosphoproteome datasets to define proteins and mechanisms responsible for the estrogen independent phenotype. While proteome and phosphoproteome varied greatly depending on protein function (e.g. RNA transcription *vs* GTPase activity), common enrichments were observed (e.g. STT3B protein levels and S498 phosphorylation). Protein abundance and phosphorylation levels are respectively dependent on (e.g.) rate of transcription and kinase activity, which in turn explain the discrepancies observed. In addition to this, the low correlation between protein abundance and phosphorylation levels of proteins involved in gene regulation might relate to the significant enrichment of proliferation pathways observed in our differential expression and pathway analyses (Figs. 4 and 5). With this in mind, we cross-referenced our proteomic datasets with previously performed CRISPR-screens^11^ and RNA-seq data^9^ to select targets for further verification. Here we observed enrichment in components of the immunoproteasome (PSMB10) in *ESR1* mutants, the expression of which is related to activation of the interferon gamma signaling pathway. Given the lack of commercial immunoproteasome inhibitors, we employed 3 inhibitors of the 20S/26S proteasome^46–48^, although no difference in growth was observed between *wt* MCF7 and *ESR1* mutants. A recent report demonstrated profound differences in the makeup of tumor associated immune cells for metastatic tumors from ER mutants compared with those from *wt* ER, including higher levels of regulatory and helper T cells and immune-suppressive macrophages^10^, raising the possibility that the elevated interferon signaling proteins identified here have an immunosuppressive role in aiding the metastatic potential of ER mutant breast cancer. On top of this, proteasome inhibition coupled with Fulvestrant treatment was tested in a phase II clinical trial, with enhanced effects in AI resistant breast cancers^40^. While we could not show that inhibition of the immunoproteasome, as part of interferon gamma signaling, had any effect on *ESR1* mutant cell growth rates over *wt* cells recent studies have revealed the activation of immune pathways through the acquisition of basal tumor features, indicating a role towards immune escape and tissue invasion rather than just proliferation^49,50^. Further investigation of these mechanisms might offer new avenues for immunotherapy regimens through in vitro co-culture experiments (e.g. tumor cells and leukocytes) and in clinical specimens (tumor and blood). This experimental venue would be able to clarify the interaction between the immune system, *ESR1* mutated breast cancers, and the signaling between these two actors.

We present here the first proteomic-centered analysis of *ESR1* mutant cells, where we characterized changes in total protein abundance and phosphorylation over estrogen deprivation and stimulation conditions. The data presented here offers a new angle on the elucidation of estrogen independent growth associated to the expression of ER mutations, constituting a resource for further investigation.

## Methods

### Cell lines and culturing

MCF7-*wt*, MCF7_Y537N_ and MCF7_Y537S_, have been previously described^9,14,25^. The T47D (wt and Y537S) were described in *Williams *et al.^10^. All cell lines were cultured in Dulbecco's modified Eagle's medium (DMEM supplemented with GlutaMAX™; Thermo-Fisher) containing 10% fetal bovine serum (FBS; Gibco) supplemented with minimal essential aminoacids (MEAA; Thermo-Fisher) and antibiotics (100 units/mL penicillin G; 0.1 mg/mL streptomycin; 45 µg/mL Gentamycin; Thermo-Fisher). For estrogen stimulation experiments, cells were cultured for 72 h in phenol red-free media supplemented with 10% charcoal-stripped FBS (csFBS; Gibco) and antibiotics (as above). Subsequently, medium was changed and E2 solubilized in ethanol was added to a final concentration of 1 nM. An equal volume of EtOH (vehicle) was added to the no ligand controls. Cells were incubated for 6 h before harvesting. When treating MCF7 and T47D cells included in the second dataset, di-methylsulfoxide (DMSO) was used as vehicle and final concentration of E2 was 10 nM. Here, harvesting was performed at 6 h and 24 h. In addition to this, full media harvests were conducted in parallel. All estrogen stimulation/deprivation experiments were conducted for 3 biological replicates.

### Cell growth assays

For cell growth assays, cells cultured in estrogen-depleted medium for 72 h were seeded into 96-well plates (Thermo-Fisher) at a concentration of 5000 cells/well (3 wells per cell line/condition in each biological replicate). After attachment (~ 12–16 h), medium was changed and supplemented with either E2 (Sigma-Aldrich) or EtOH as vehicle. The Sulphorhodamine B (SRB) assay was then used to determine cell numbers^9^. Briefly, cells cultured in 96-well plates were fixed by adding 100 μL/well of 40% trichloroacetic acid (Sigma) and incubated at 4 °C for 1 h. Plates were washed six times with distilled water and air-dried. Total cellular protein was stained by adding 100 μL/well of 0.4% (w/v) Sulphorhodamine B—SRB (Sigma) in 1% acetic acid (VWR) and incubated at RT for 1 h. Plates were washed 6 times with 1% V/V acetic acid to remove the unbound dye and air-dried overnight. A volume of 100 μL of 10 mM Tris was added to solubilize the SRB dye in each well. Absorbance was measured at 492 nm wavelength in a microplate reader (Sunrise, Tecan).

For cell growth inhibition assays, cells were seeded at 2000 cells/well in 96-well plates. After 48 h, cells were treated with an increasing concentration of Everolimus (MedChemExpress), Palbociclib (Sigma-Aldrich), Bortezomib (Tocris), Carfilzomib (Tocris), Ixazomib (MedChemExpress), and Fulvestrant (MedChemExpress). Drug stock solutions prepared in DMSO were added to culture media at 1:1000 dilution. An equal volume of DMSO was added as vehicle control at a final concentration of 0.1%. Following five days of treatment, cell growth was measured by SRB assay. Growth inhibition dose–response curves and half-maximal inhibitory concentrations (IC50) were calculated by GraphPad Prism (v9.2.0) using non-linear regression models. Assessment of synergistic drug effects out of dual drug treatment experiments, SynergyFinder + ^51^ was employed using the Bliss model^52^.

### Rapid Immunoprecipitation of Endogenous Proteins

For rapid immunoprecipitation of endogenous proteins (RIME), cells were cultured in the full media, then processed according to established protocols^53^. Briefly, protein complexes were cross-linked using formaldehyde (1% v/v in culture medium) for 10 min; reactions were quenched with 1 M Glycine solution, and cells washed 3 times with DPBS before harvesting. Nuclear and cytosolic fractions were separated by centrifugation after re-suspension of cell pellets in Lysis Buffer 1 (10% v/v glycerol, 0.5% v/v NP40 substitute, 0.25% v/v TritonX-100, 1 mM EDTA, 140 mM NaCl in 50 mM HEPES pH 7.5). Nuclear fractions (precipitates) were re-suspended in Lysis Buffer 2 (1 mM EDTA, 0.5 mM EGTA, 100 mM NaCl in 10 mM Tris pH 8.0), centrifuged, re-suspended in Lysis Buffer 3 (0.1% w/v sodium deoxycholate, 0.5% w/v N-lauroylsarcosine, 1 mM EDTA, 0.5 mM EGTA, 100 mM NaCl in 10 mM Tris pH 8.0), and sonicated (20 cycles: 30 s ON, 30 s OFF; Bioruptor, Diagenode). Triton X-100, to a final concentration of 1% V/V, was added to lysates, centrifuged at ~ 20,000×*g* and supernatants were incubated with an ER antibody (sc-8002 Santa Cruz) or IgG2a (ab18413 Abcam) as a negative control, previously conjugated with protein A magnetic beads (Thermo-Fisher). Lysates were incubated overnight at 4 °C under agitation, then washed 10 times with cold wash buffer (0.7% w/V sodium deoxycholate, 1% V/V NP40 substitute, 1 mM EDTA, 500 mM lithium chloride in 50 mM HEPES pH 7.6), twice with 100 mM ammonium bicarbonate buffer, and proteins were digested on beads using trypsin (first digestion: 37 °C overnight incubation; second digestion: 37 °C 4 h incubation). Peptide mixtures were then acidified using a 5% formic acid (FA) solution.

### Cell lysis and trypsin digestion for proteome and phosphoproteome analyses

E2 and vehicle (EtOH or DMSO) treated cells for MS experiments were harvested in cold DPBS, pelleted, and re-suspended in RIPA buffer supplemented with protease and phosphatase inhibitors (Halt Protease Inhibitor and 0.5 M EDTA; Thermo-Fisher). Cells were sonicated for 20 min (20 cycles: 30 s ON, 30 s OFF; Bioruptor, Diagenode), lysates were spun down at 14,000×*g* for 30 min at 4 °C, and supernatants were collected. Protein concentration was measured with the bicinchoninic acid assay (Thermo-Fisher), according to manufacturer’s instructions. A total of 100 µg and 500 µg of proteins were digested for downstream proteome and phosphoproteome analyses, respectively as previously described^54^. Briefly, proteins were precipitated using ice-cold methanol and spun down at 14,000×*g* for 20 min at 4 °C, supernatants were discarded. Pellets were then re-suspended in 100 mM Tris buffer containing 100 mM dithiothreitol and 4% w/V sodium-dodecyl-sulphate (pH 8.0), heated at 95 °C for 30 min under mild agitation, then in 8 M urea in 100 mM Tris buffer (pH 8.0). Urea re-suspended samples were loaded on 30 KDa molecular filters (Millipore) and centrifuged at 14,000×*g* for 20 min. Filters were then twice washed with 8 M urea buffer and incubated in 50 mM iodoacetamide in 8 M urea buffer for 30 min (in the dark). Filters were washed 4 times (2 × 8 M urea buffer, 2 × 50 mM tri-ethyl-ammonium bicarbonate buffer pH 8.0), and proteins were digested with trypsin (enzyme-protein ratio 1:50) at 37 °C for 16 h under agitation (650RPM). Filters were then centrifuged at 14,000×*g* for 20 min to extract tryptic peptides.

The second set of MCF7 and T47D lysates was prepared as above, with modifications. Precipitated proteins were re-suspended in 8 M urea in 100 mM ammonium bicarbonate buffer (pH 8.0), reduced with 50 mM tris(2-carboxyethyl)phosphine, alkylated with 50 mM iodoacetamide, and digested with trypsin overnight at 37 °C for 16 h under agitation.

### Peptide fractionation and phosphopeptide enrichment

Tryptic peptide mixtures were fractionated for downstream MS analysis using strong anion exchange (SAX) and immobilized-ion affinity columns (IMAC) for proteome and phosphoproteome analyses, respectively.

SAX fractionation was performed as previously described^17^. Briefly, peptides mixtures were dried and re-suspended in Britton and Robinson Universal Buffer (BRUB; 20 mM phosphoric acid, 20 mM boric acid and 20 mM acetic acid in ultrapure water; BRUB) pH 11 and loaded on SAX (Sigma) stage tips combined with C18 filters (Sigma). SAX filters-containing tips were used to elute peptides onto C18 tips (Sigma) using BRUB at decreasing pH: 8, 6, 5, 4, and 3. C18 tips were then washed with 0.1% FA solution, and peptides eluted with 0.1% FA and 80% acetonitrile (ACN) in ultrapure water. SP3 peptide purification was subsequently performed on dried eluates as in previously published protocols^55^. SP3 beads (Thermo-Fisher) were added to peptides, peptides were captured by adding a volume of 200 µL of ACN and eluted with 2% dimethyl sulfoxide in water. Supernatants were dried and stored at – 80 °C until MS analysis.

IMAC phosphopeptide enrichment was performed by sequentially using the High-Select™ TiO_2_ and Fe-NTA kits (Thermo-Fisher). Prior to enrichment, peptide mixtures were de-salted using Oasis HLB columns (Waters). Briefly, sorbents were conditioned and equilibrated with 2 washes (each) of 100% V/V methanol, 80% ACN and 0.1% in ultrapure water, and 0.1% FA in water. Samples were then loaded twice to maximize recovery, washed 4 times with 0.1% FA in water, and eluted with 45% ACN and 0.1% FA in water. Eluates were dried in a concentrator. RIME-derived peptide mixtures were also de-salted using this method. Phosphopeptide purification was performed according to manufacturer instructions, where washing step eluates from the TiO_2_ enrichment step were loaded on the Fe-NTA ones. Eluates from both kits were dried and stored at – 80 °C until MS analysis.

Peptides from MCF7 and T47D cells were labeled using TMT6plex (Thermo Scientific) and fractionation was carried on using high-pH spin columns (Thermo Scientific) according to manufacturer instructions. About 95% of each eluate was further processed for TiO_2_-based phosphopeptide enrichment, while the remaining 5% was employed for proteome analysis. Peptide and phosphopeptide fractions were desalted as above.

### Mass spectrometry analysis

RIME peptide mixtures were analyzed on a Q-Exactive Plus, coupled to a nano LC Easy system (Thermo-Fisher). Peptides were separated on an EasySpray HPLC column (ID 75 µm × 25 cm C18 2 µm, 100 Å resin; Thermo-Fisher) in a 110 min gradient (flow: 300 nL/min; mobile phase A: 0.1% formic acid in H_2_O; mobile phase B: 100% acetonitrile and 0.1% formic acid). The gradient was run as follows: 5% B for 5 min; 5–30% B in 90 min; 30–95% B in 5 min; 95% B for 10 min. The 15 most abundant peaks from the MS scan (resolution: 70,000 at 200 m/z) were selected and fragmented by higher energy induced collision dissociation (HCD; collision energy: 30). Automatic Gain Control (AGC) target for full MS and MS/MS scans was set to 1E6.

Global proteome MS analysis was performed on a Q-Exactive HF-X (Thermo-Fisher) mass spectrometer. Around 1 µg of Tryptic peptides from fractionated samples were first trapped on an Acclaim PepMap™ 100 pre-column (ID 75 µm × 2 cm C18 3 µm, 100 Å resin; Thermo-Fisher), and separated on a reverse phase HPLC EasySpray column (ID 75 µm × 50 cm C18 2 µm, 100 Å resin; Thermo-Fisher) coupled to an Dionex Ultimate 3000 liquid chromatography system (Thermo-Fisher). Peptides were eluted in a 120 min gradient (flow: 350 nL/min; mobile phase A: 0.1% formic acid in H2O; mobile phase B: 80% acetonitrile and 0.1% formic acid). The chromatographic gradient was run as follows: 10% B for 4 min; 10–30% B in 86 min; 30–45% B in 20 min; 45–95% B in 1 min; 95% B for 9 min. The 15 most abundant peaks from the MS scan (resolution: 60,000 at 200 m/z) were selected and fragmented by higher energy induced collision dissociation (HCD; collision energy: 28). Dynamic exclusion was activated (window: 10 s) Automatic Gain Control (AGC) target for full MS and MS/MS scans was set to 3E6 and 1E5, respectively.

As with the proteome runs, phosphoproteome peptide mixtures were analyzed on a Q-Exactive HF-X (Thermo-Fisher) system coupled to a nano-LC easy 1200 chromatography system (Thermo-Fisher). Trapping, analytical columns, mobile phases and flow employed in this analysis were the same as above. Gradient was as follows: 10–30% B in 90 min; 30–45%B in 20 min; 45–95% B in 1 min; 95%B for 9.5 min. Full MS scan parameters: 60,000 resolution, AGC target was set to 3E6. MS/MS scan parameters: 15,000 resolution, AGC target was set to 1E5. Collision energy was set to 28, with 15 analyzed peaks and a 10 s dynamic exclusion window.

TMT-pools were analyzed on a Q-Exactive HF-X (Thermo-Fisher) system coupled to a nano-LC easy 1200 chromatography system (Thermo-Fisher). Trapping, analytical columns, mobile phases and flow employed in this analysis were the same as above. Gradient was as follows: 5–25% B in 110 min; 25–40%B in 10 min; 40–95% B in 10 min; 95%B for 0.5 min. Full MS scan parameters: 120,000 resolution, AGC target was set to 3E6. MS/MS scan parameters: 45,000 resolution, AGC target was set to 1E5. Collision energy was set to 34, with 20 analyzed peaks and a 30 s dynamic exclusion window.

### Mass spectrometry data processing

MS analysis-derived RAW files were analyzed using MaxQuant^56^ (v1.6.14.0), and MS spectra searched using the Andromeda^57^ search engine with the Uniprot-Swissprot human proteome database (version download: 2020.02.24; second dataset: 2022.04.02). For RIME analysis MaxQuant (v1.5.5.1) was employed and modified *ESR1* peptide sequences were collated, generating a new FASTA file (from Uniprot version download: 2017.06.23). Selected protease was Trypsin. Carbamidomethylation of Cys residues was selected as fixed modification (set as variable modification for RIME data search), while Met oxidation and acetylation of N-terminal residues were selected as variable ones. For the phosphopeptide search, phosphorylation of Ser/Thr/Tyr residues was additionally selected as variable modification, while Label-free Quantification (LFQ) was activated for the proteome data search. Identification of peptides resulting from missed cleavages was allowed. Precursor ion tolerance: 20 and 4.5 ppm for first and main searches, respectively. Match-between-run option was enabled and settings left to default. External dataset proteome files^14,22^ were searched using the same settings as our proteome dataset, but enabling dimethyl and Tandem Mass Tag settings, respectively. For analysis of TMT pools, MS2 reporter ion options were enabled and TMT6plex was selected. Filtering on minimum reported precursor intensity fraction was set to 0.75. Other parameters were set as above.

Protein and phosphorylated peptide intensity files (label-free and TMT) were employed for downstream proteome and phosphoproteome searches, respectively. The protein abundance table was filtered for protein q-value (< 0.01), contaminant (excluded), reverse sequences (excluded), unique peptides (at least 1). The phosphopeptide table was additionally filtered for localization probability (> 0.75) of phosphorylations.

### Clinical tumor cohort

A set of 28 primary breast tumors with detected *ESR1* mutation and 108 control cases were derived from the SCAN-B tumor cohort^31^. Control cases were selected based on ER status (positive; cutoff 10% of positive cells), PgR status (positive; cutoff 10% of positive cells), adjuvant endocrine treatment, no ERBB2 amplification, and Luminal (A or B) subtype. This resulted in a final set of 26 cases and 81 controls. Gene expression in Transcripts per Kilobase Million (TPM) were employed for pathway analysis using GSEA.

### Statistical analysis

All datasets (i.e. protein LFQ intensities and phosphopeptide intensities) were Log2 transformed and filtered for missing data (30% missing data allowed across the dataset and at least 2 out of 3 replicate observations in at least one model/condition). Missing data were imputed to zero. Statistical metrics (Spearman Rho) and tests (Welch corrected t test) were calculated in R (v 4.0.3) and GraphPad (v 9.0.0), respectively. Benjamini–Hochberg *p-value* adjustment was applied to correct for multiple testing for all tests.

The TMT dataset was filtered for missing data (50% missing data allowed) and imputed using values from a normal distribution using Perseus^58^.

Analyses for differential pathway enrichment between conditions and cell models were performed using Gene Set Enrichment Analysis (GSEA)^27^; database: Hallmarks (v 5.2) and Reactome (v 5.2); permutation type: gene set; scoring: classic; metric: t test. Other parameters were kept to default settings. False Discovery Rate (FDR) cutoff to call significant pathways was set to 0.25. Normalized enrichment scores were plotted to define enrichment levels. Enrichment maps were generated in Cytoscape (v3.8.2) using GSEA output.

Kinase-Substrate Enrichment Analysis (KSEA) for all model and condition comparisons were performed in R (KSEAapp, v 0.99.0); database: PhosphoSitePlus; NetworkKIN predictions allowed (score cutoff: 5). Multiple phosphorylation on target peptides were included. *p-value* cutoff to call significantly enriched kinases was set to 0.05. Z-scores were used to define kinase enrichments.

In our correlation analyses between protein and phosphosite abundances, we employed the Spearman method to calculate both the correlation coefficient and *p-value*. To assess whether specific protein clusters were affected by different correlation distributions, all proteins were annotated with Gene Ontology Biological Process (GOBP) terms using BiomaRt (v 2.46.1). The distribution of correlation coefficients of each GOBP annotation was then tested against the background (i.e. all proteins) by t test. *p-values* were adjusted using the Benjamini–Hochberg method. The selected adjusted *p-value* cutoff for GOBP annotation was 0.05.

Druggable proteome protein list (i.e. FDA drug targets) was downloaded from proteinatlas.org. *ESR1 bona fide* interactor list was derived from^22^, while a list of *ESR1* responsive genes was derived from ENCODE Transcription Factor Targets (http://amp.pharm.mssm.edu/Harmonizome/gene_set/ESR1/ENCODE+Transcription+Factor+Targets).

### Ethical approval

The use of RNAseq data from patients of the SCAN-B cohort was approved by the Regional Ethical Review Board of Lund with diary numbers 2007/155, 2009/658, 2010/383, 2012/58, and 2013/459.

### Supplementary Information

Supplementary Figures.

Supplementary Table S1.

Supplementary Table S2.

Supplementary Table S3.

Supplementary Table S4.

Supplementary Table S5.

Supplementary Table S6.

Supplementary Table S7.

Supplementary Table S8.

Supplementary Table S9.

Supplementary Table S10.

Supplementary Table S11.

Supplementary Table S12.

Supplementary Table S13.

## Data Availability

Proteomic data and related sample descriptor files have been deposited to the ProteomeXchange Consortium via the PRIDE partner repository^59^ with dataset identifiers PXD032369 (proteome) and PXD032285 (phosphoproteome). RIME data from full media culture was uploaded with identifier PXD032404. The TMT dataset including MCF7 and T47D (proteome and phosphoproteome) cells was uploaded with identifier: PXD046446.
