# Supplementary material for: Proteomic profiling reveals that ESR1 mutations enhance cyclin-dependent kinase signaling
Source: Sci Rep. 2024 Mar 22;14:6873. doi: 10.1038/s41598-024-56412-8 (PMC10959978; doi:10.1038/s41598-024-56412-8)
Supplement: Supplementary file 1 — Supplementary Figures. [file 41598_2024_56412_MOESM1_ESM.docx]

**TITLE: “Proteomic profiling reveals that *ESR1* mutations enhance cyclin-dependent kinase signaling”**

**Authors**

Tommaso De Marchi^1^, Chun-Fui Lai^2^, Georgia M. Simmons^2^, Isabella Goldsbrough^2^, Alison Harrod^2^, Thai Lam^1^, Laki Buluwela^2^, Sven Kjellström^3,4^, Christian Brueffer^5^, Lao H. Saal^5^, Johan Malmström^6^, Simak Ali^2^ & Emma Niméus^1,7^

**Author affiliations**

1. Division of Surgery, Oncology, and Pathology, Department of Clinical Sciences, Lund University, Solvegatan 19, SE 22362, Lund, Sweden.

2. Department of Surgery & Cancer, Imperial College London, Hammersmith Hospital Campus, Du Cane Road, W12 0NN, London, United Kingdom.

3. Department of Biochemistry and Structural Biology, Center for Molecular Protein Science, Lund University, Solvegatan 19, SE 22362 Lund, Sweden.

4. Swedish National Infrastructure for Biological Mass Spectrometry – BioMS

5. Division of Oncology, Department of Clinical Sciences Lund, Lund University, Medicon Village, SE 22381, Lund, Sweden.

6. Division of Infection Medicine, Department of Clinical Sciences Lund, Faculty of Medicine, Lund University, Klinikgatan 32, SE 22184, Lund, Sweden.

7. Department of Surgery, Skåne University Hospital, Lund, Sweden.

**Supplemental Figures and Legends**


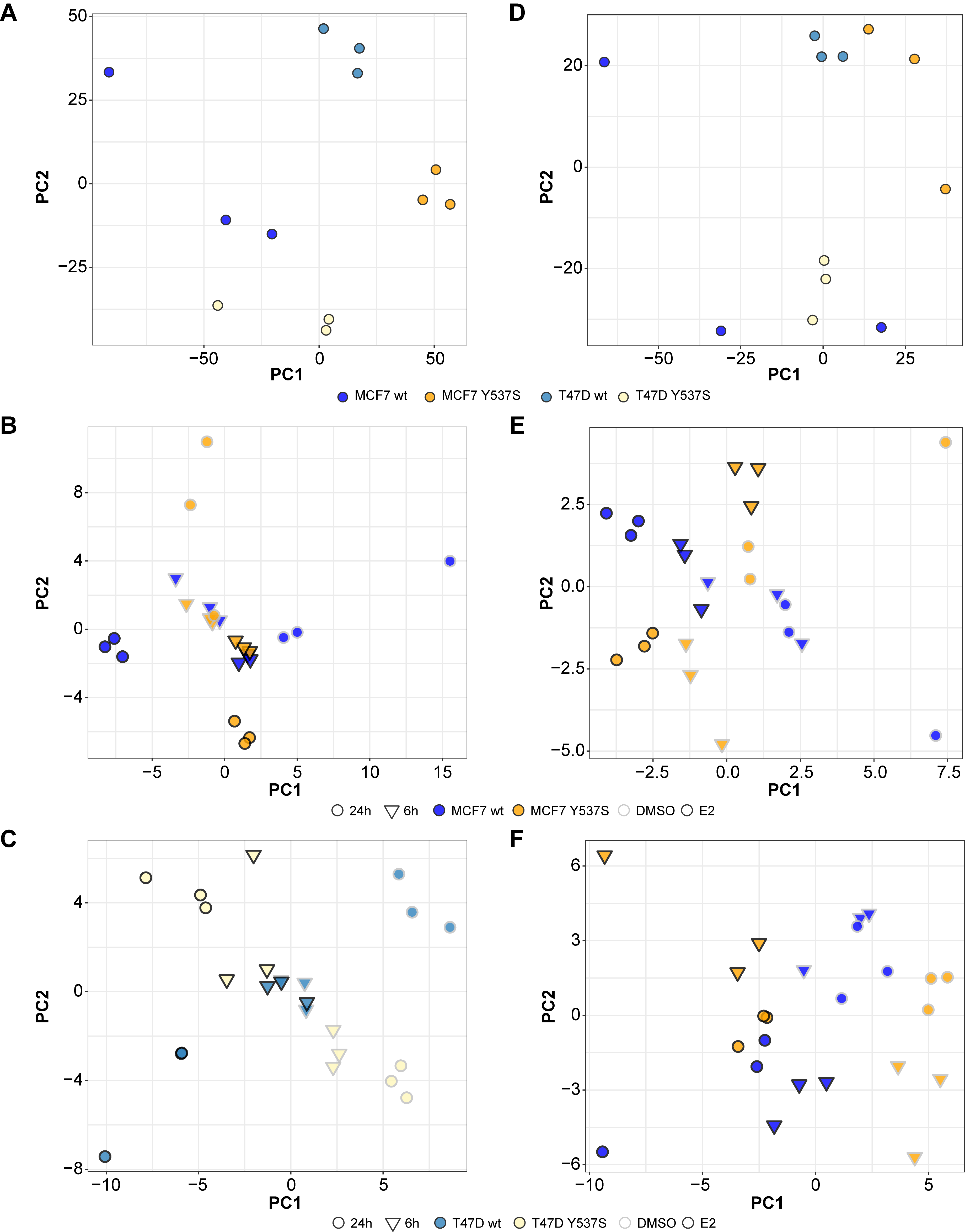


**Figure S1. Principal component analyses of additional dataset.**

In addition to our initial label-free proteomic dataset we analyzed a second set of cells comprising MCF7 and T47D models harboring the *ESR1* Y537S mutation (see *Methods* for details). This integrated proteomic (panels A-C) and phosphoproteomic (panels D-F) dataset comprised cells cultured in full medium as well as after estrogen deprivation or stimulation. Panels A-C depicts PCA results for cells grown in full medium, MCF7 cells harvested at 6h and 24 after estrogen stimulation or deprivation, and T47D treated cells at 6h and 24h time points, respectively. Panels D-F represents the same analyses for the phosphoproteomic part of the dataset.

Acronyms: PCA: principal component analysis.


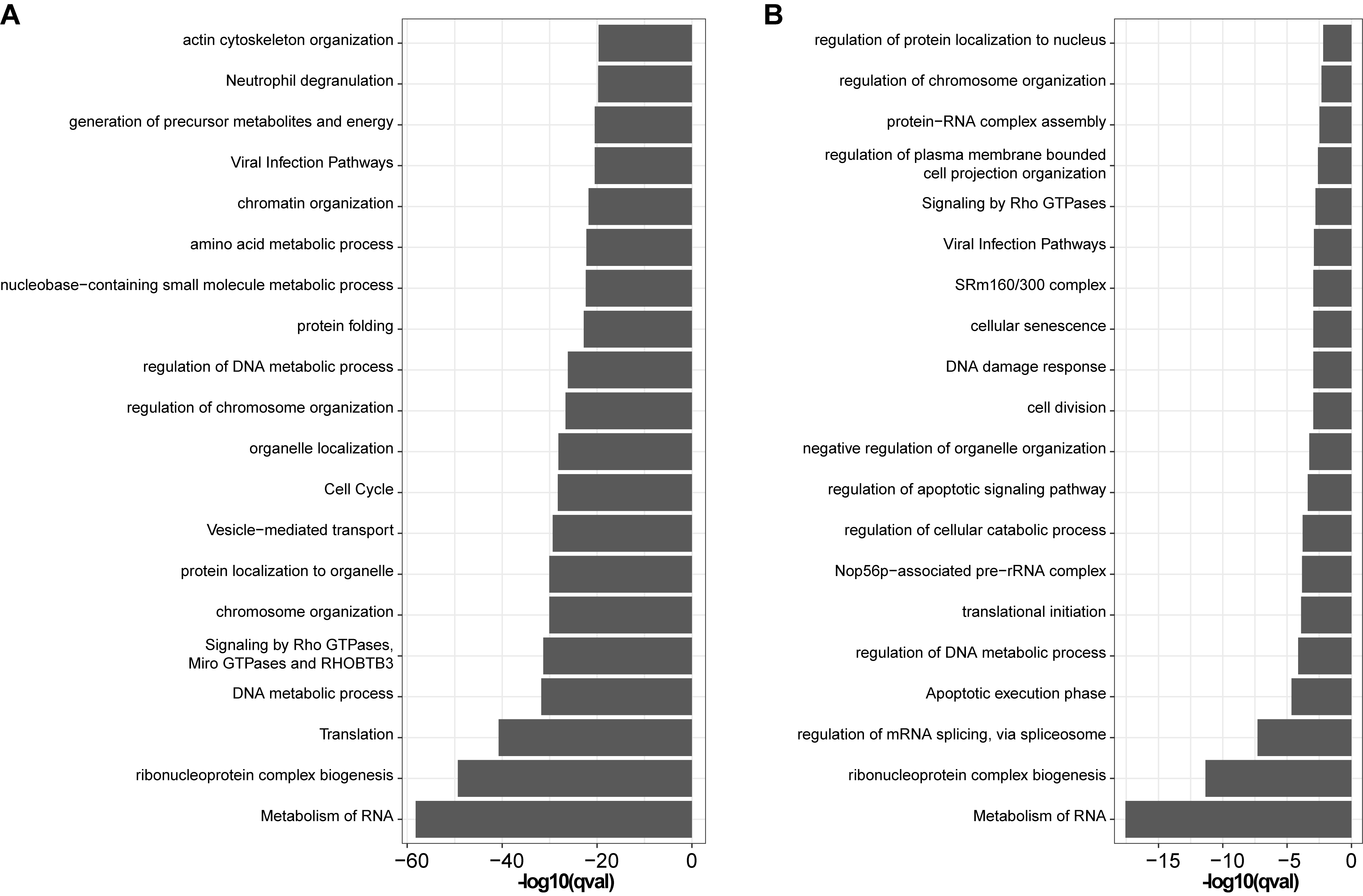


**Figure S2. Expression of lobular-specific genes.**

Panels A and B display metascape (Zhou *et al*, 2019) analyses proteins and proteins matching to phosphosites selected based on ANOVA test significance between the MCF7 and T47D subsets of our additional dataset.

Acronyms: ANOVA: analysis of variance.


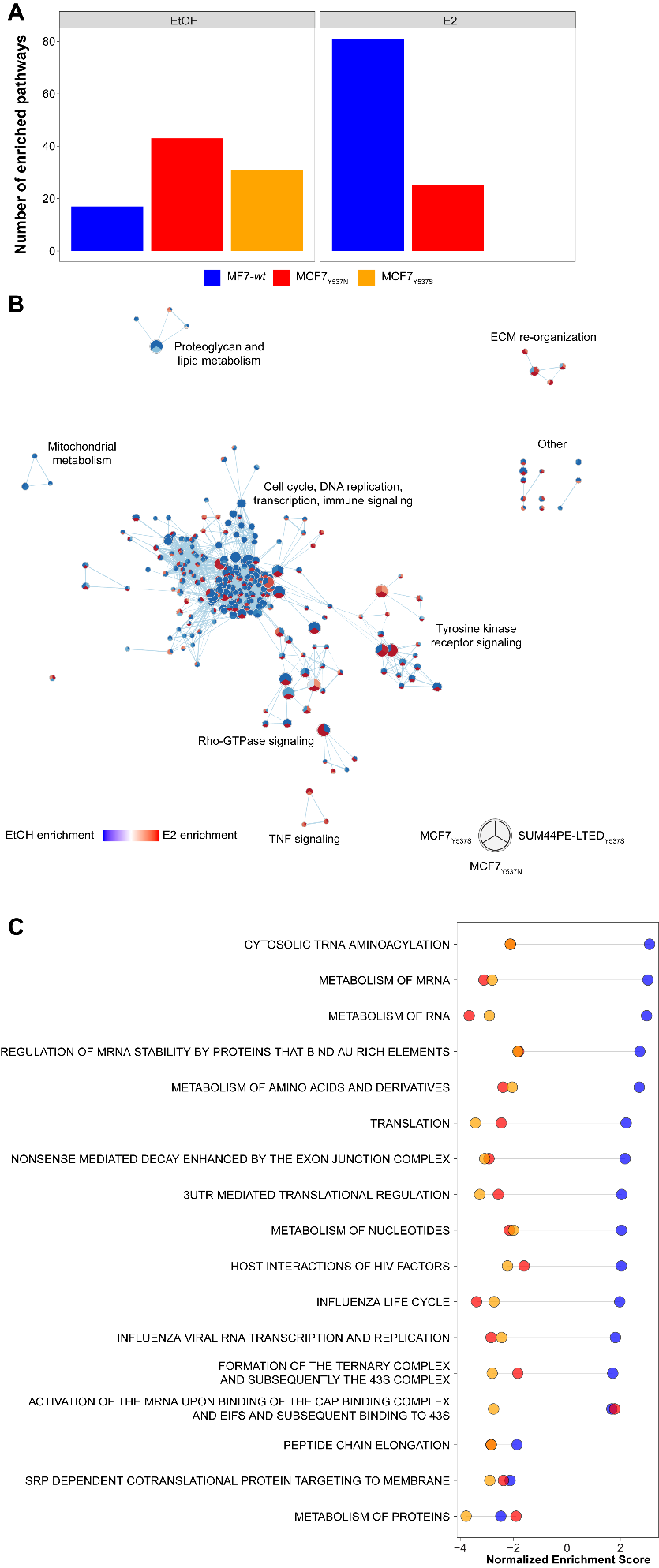


**Figure S3. REACTOME-based enrichment maps in response to estrogen stimulation.**

GSEA analysis was performed on each cell line (i.e. MCF7-*wt*, MCF7_Y537N_, and MCF7_Y537S_), comparing estrogen deprivation (EtOH) and stimulation (E2) conditions. The union of significant pathways (FDR < 0.25) was then plotted using the Enrichment map plugin in Cytoscape (REACTOME pathways only). Panel A displays the number of enriched REACTOME pathways for each cell line across conditions. Enrichment is represented in panel B. The overlap of significantly enriched pathways (FDR < 0.25) out of GSEA analyses (E2 vs EtOH; REACTOME database) for each model are depicted in panel C bubble plot. Dot colors represent each model. Positive enrichment score refers to enrichments after E2 stimulation, while negative ones to enrichments after E2 deprivation

Acronyms: E2: 17-β-estradiol; ER: estrogen receptor; EtOH: ethanol; GSEA: gene set enrichment analysis.


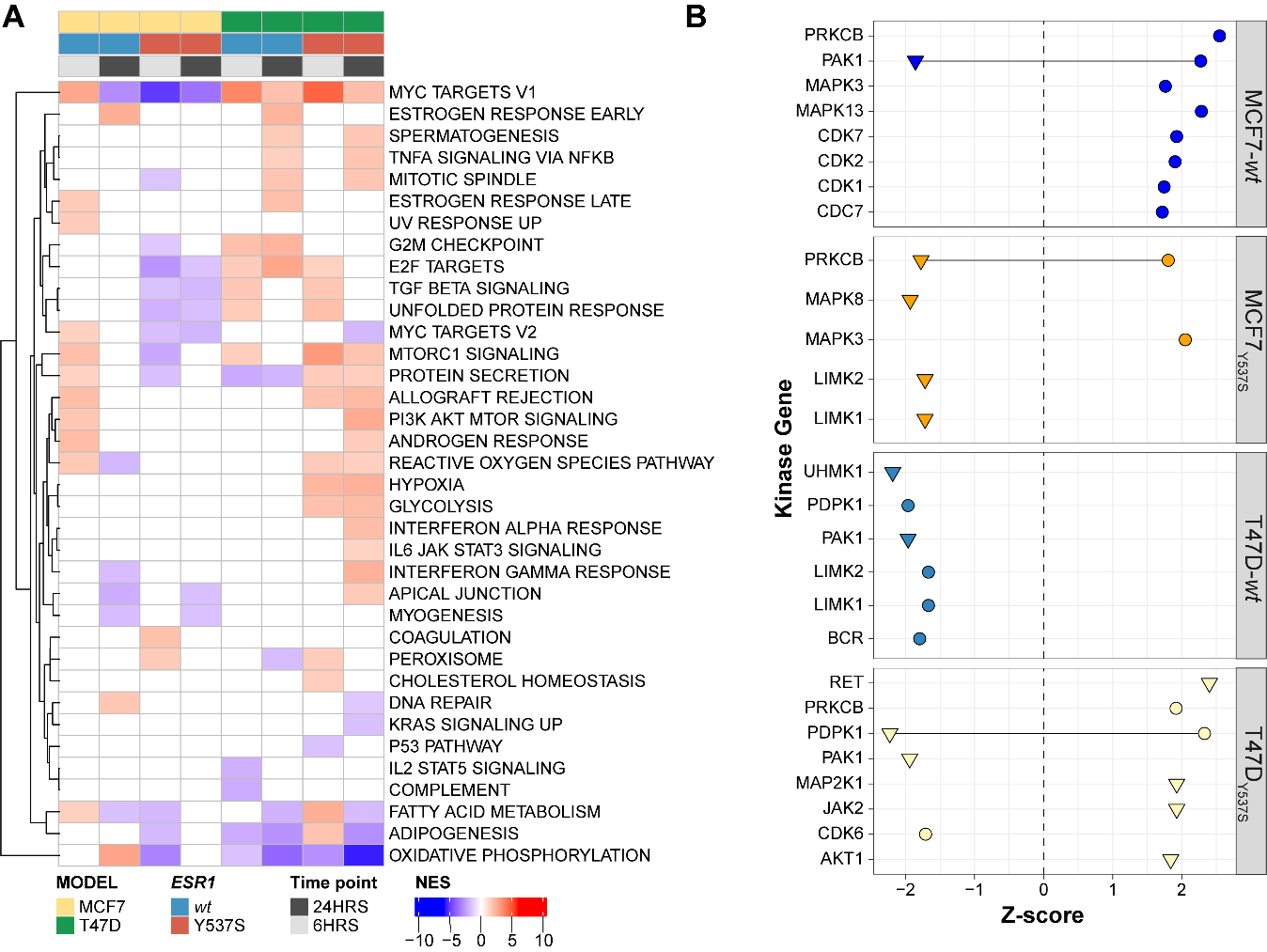


**Figure S4. Response to estrogens in the additional MCF7 and T47D dataset.**

GSEA and KSEA analyses were performed on MCF7 and T47D cells (bearing *wt* and Y537S mutated *ESR1*) comparing estrogen stimulation and deprivation treatments. Significant pathways (GSEA) and Kinase enrichments (KSEA) are depicted in panel A and B, respectively. In panel B, positive Z-score represents E2 enrichment, while negative score represents enrichment under estrogen deprivation.

Acronyms: E2: 17-β-estradiol; ER: estrogen receptor; EtOH: ethanol; MS: mass spectrometry; GSEA: gene set enrichment analysis; KSEA: kinase substrate enrichment analysis.


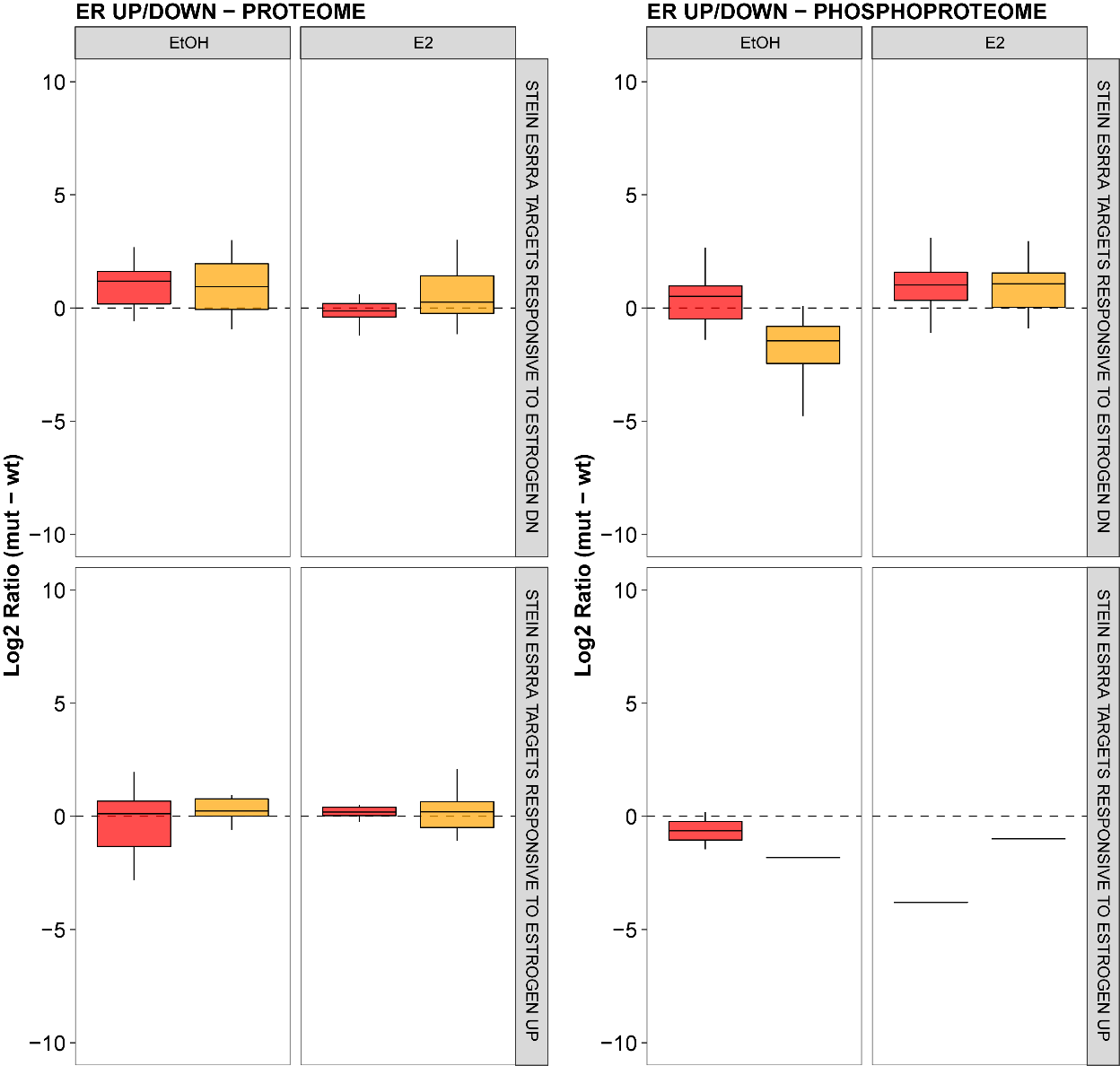


**Figure S5. Enrichment of upregulated and downregulated genes in *ESR1* mutants.**

Boxplots display enrichment of genes responsive to *ESR1* activation in mutant cells over *wt*.

Acronyms: E2: 17-β-estradiol; ER: estrogen receptor; EtOH: ethanol.


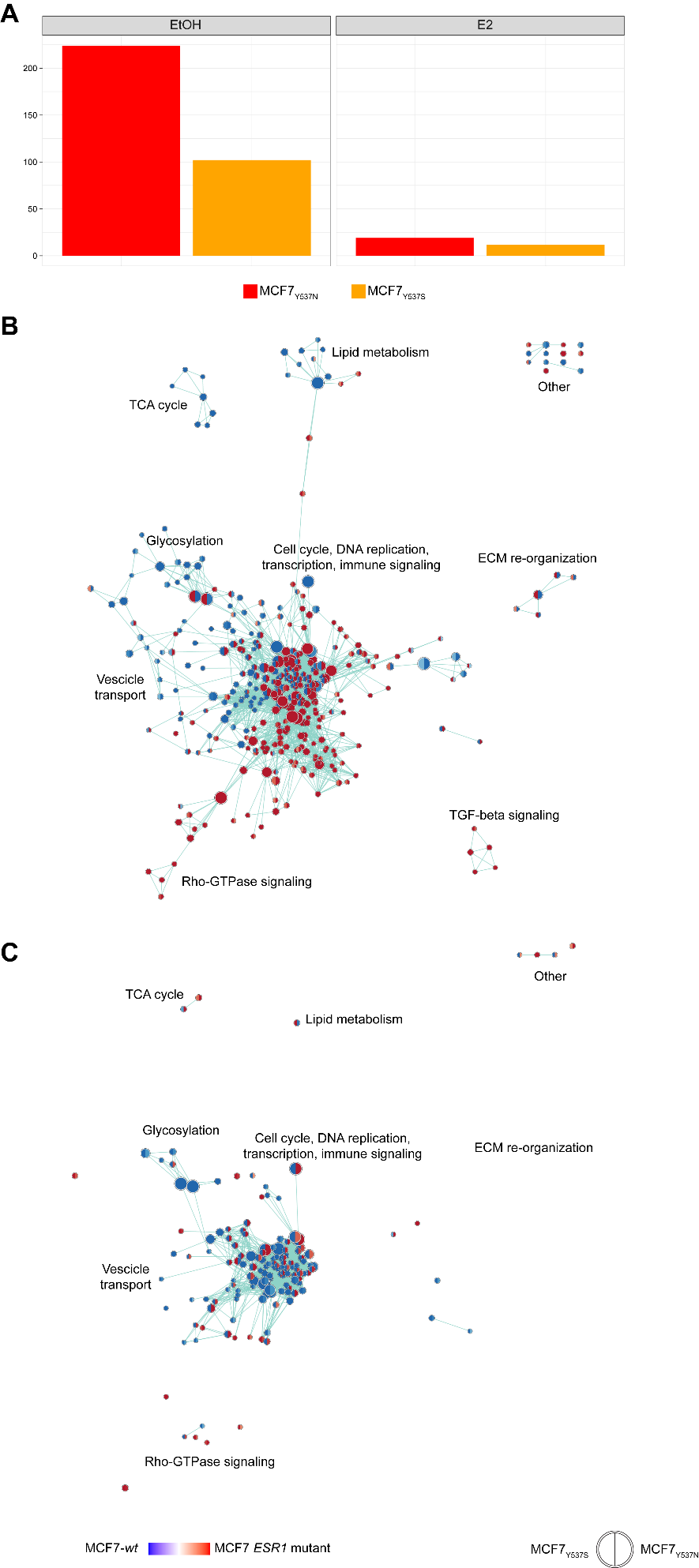


**Figure S6. REACTOME-based enrichment maps of *ESR1* mutants over *wt* cells.**

GSEA analysis was performed for each *ESR1* mutant model (MCF7_Y537N_, and MCF7_Y537S_) and compared with its *wt* counterpart, under E2 and vehicle (db: REACTOME). The union of significant pathways (FDR < 0.25) was then plotted using the Enrichment map plugin in Cytoscape (REACTOME pathways only). Panel A displays the number of enriched REACTOME pathways for each cell line across conditions. Enrichment maps for vehicle and estrogen conditions comparing mutants to *wt* cells are displayed in panels B and C, respectively.

Acronyms: E2: 17-β-estradiol; ER: estrogen receptor; EtOH: ethanol; GSEA: gene set enrichment analysis.


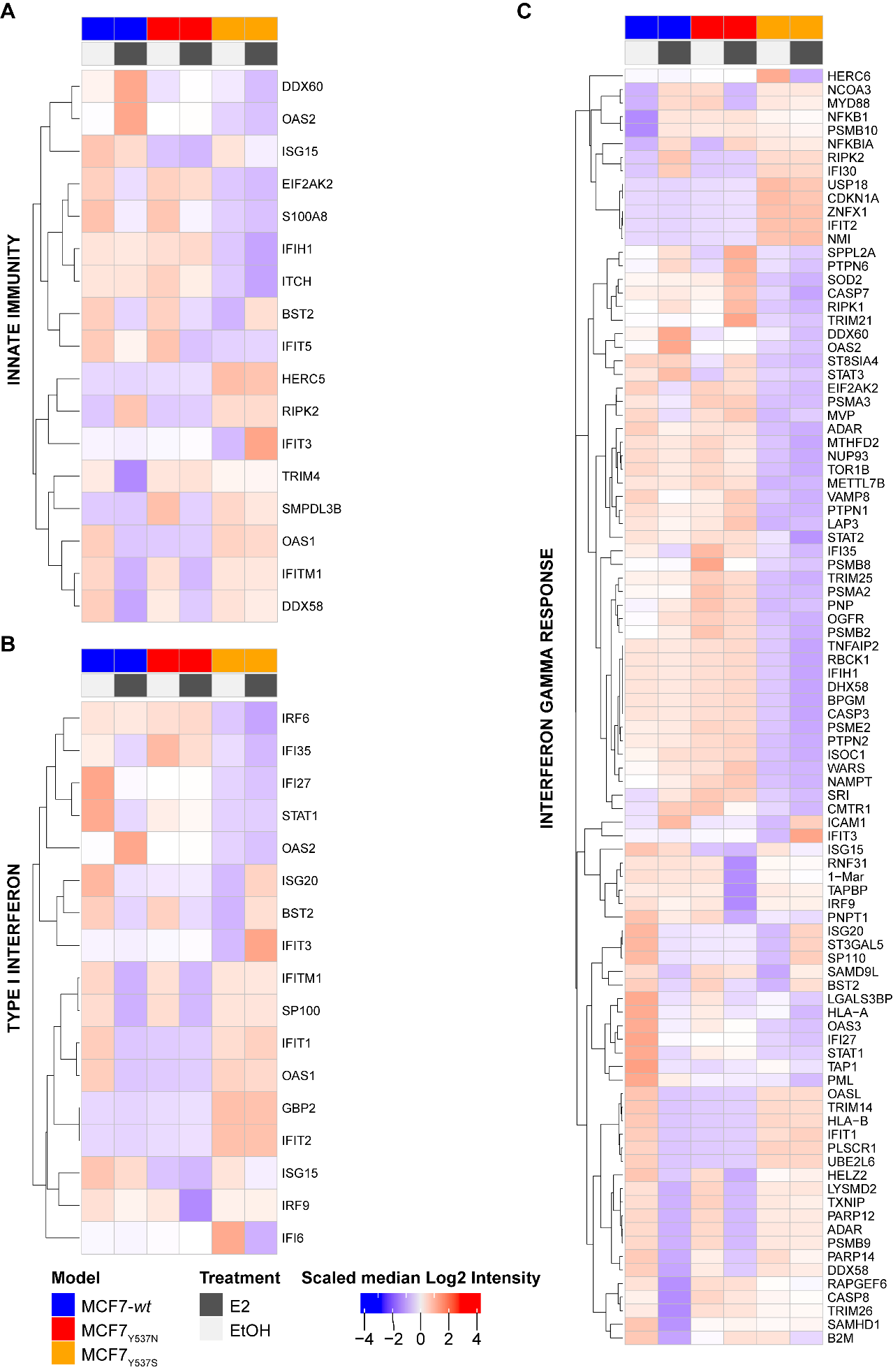


**Figure S7. Expression of immune signature genes in the proteomic dataset.**

Protein expression levels of immune signaling pathways (out of DAVID analyses reported in Williams *et al*, 2020) were plotted in heatmaps. Panel A: Innate Immunity; panel B: Type I Interferon. These pathways overlapped with the INTERFERON GAMMA RESPONSE gene set out of GSEA analyses, which is displayed in panel C.

Acronyms: E2: 17-β-estradiol; ER: estrogen receptor; EtOH: ethanol; GSEA: gene set enrichment analysis.


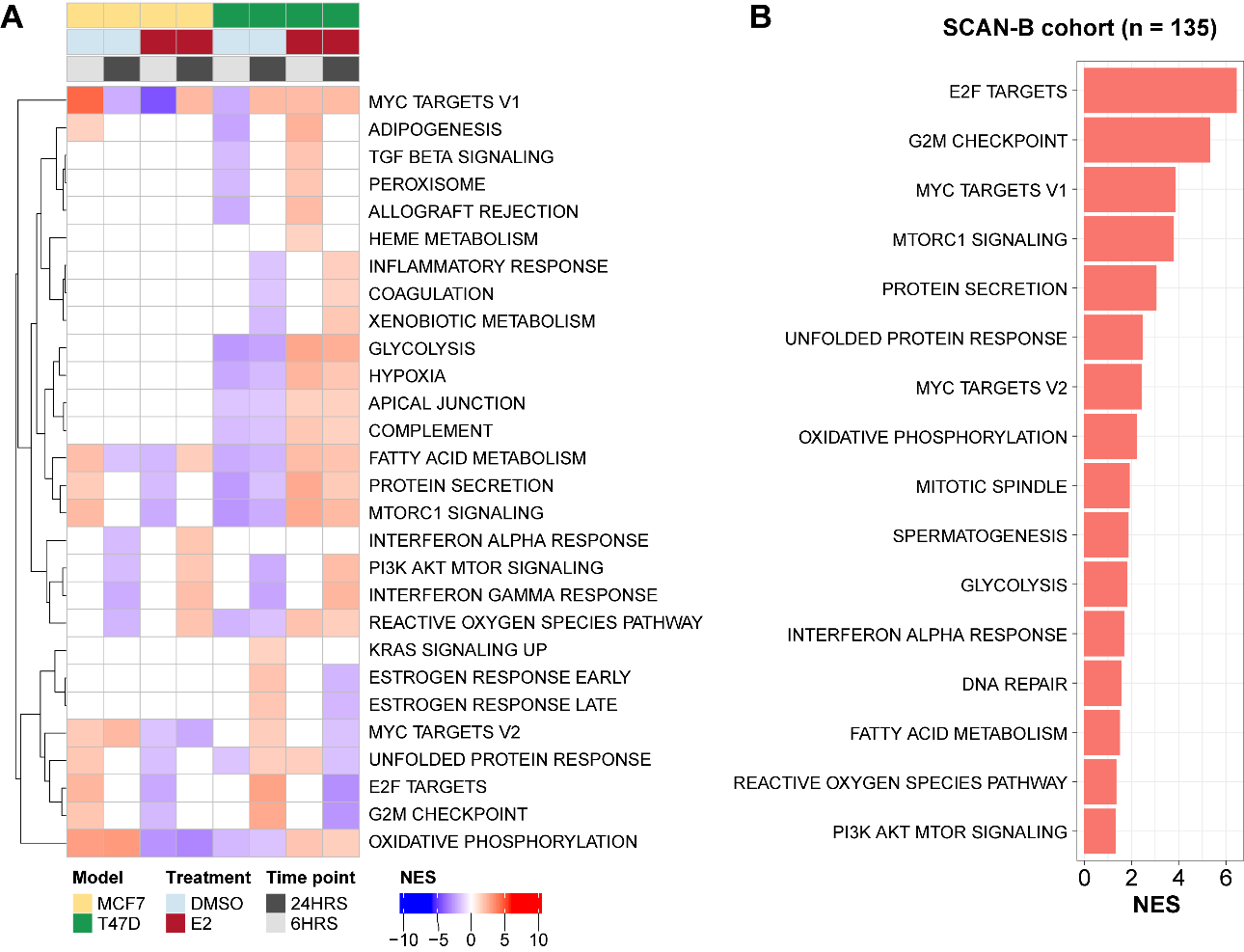


**Figure S8. ESR1 mutant gene set enrichments in pre-clinical and clinical datasets.**

GSEA analysis was performed on MCF7 and T47D cells (bearing *wt* and Y537S mutated *ESR1*) comparing each mutant to its isogenic counterpart across treatments and time points (i.e. 6h and 24h). Significant pathways are depicted in panel A. Here, positive normalized enrichment scores represents *ESR1* mutant enrichment, while negative score represents enrichment in *wt* cells. GSEA analysis was performed on RNAseq data from a clinical cohort of primary breast cancers with identified *ESR1* mutations (see *Methods* for details). Bar chart displaying gene sets enriched in *ESR1* mutant cancers are shown in panel B.

Acronyms: GSEA: gene set enrichment analysis..


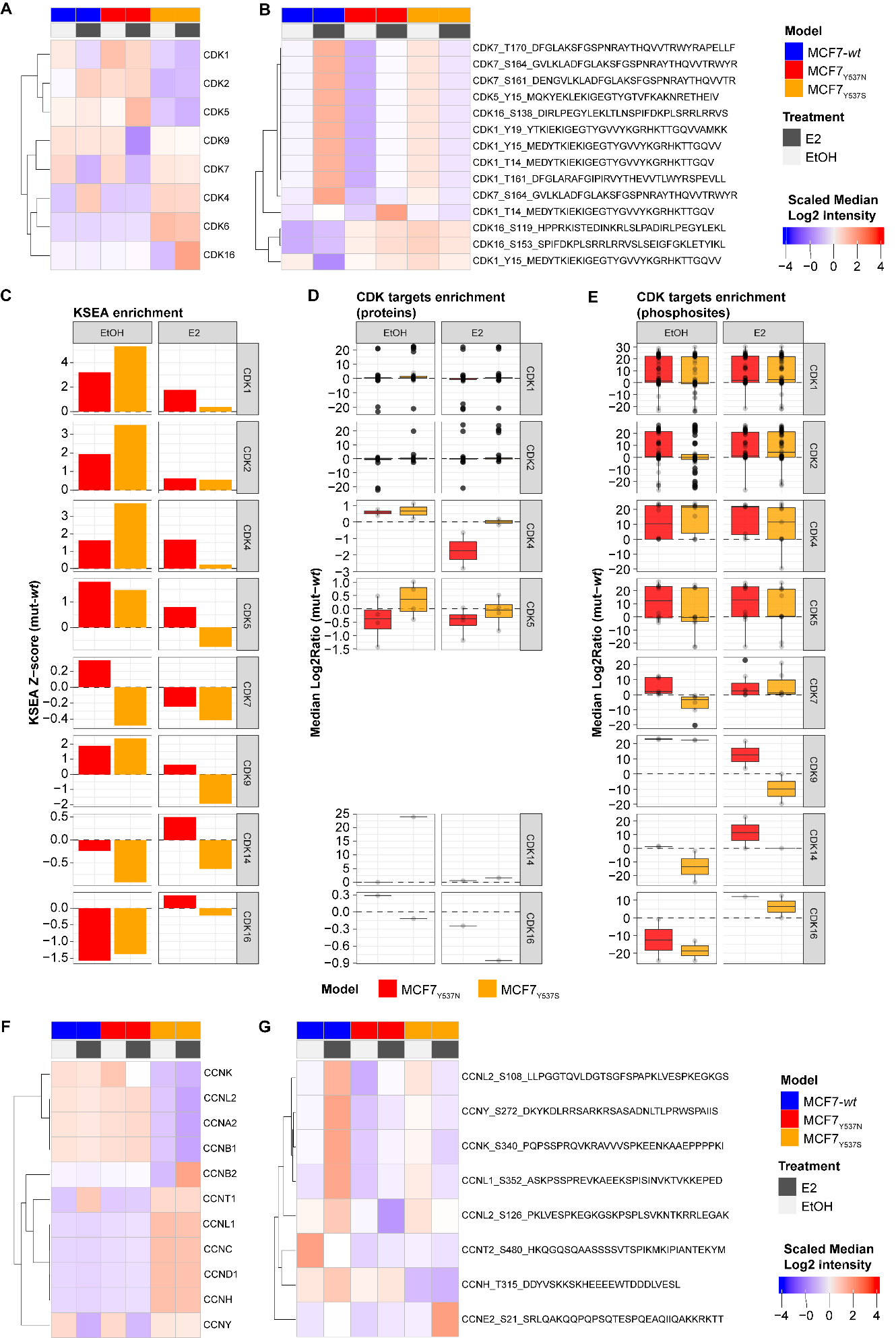


**Figure S9. Integrated analysis of CDK enrichment in our MCF7 dataset.**

Panel A displays heatmap of proteome-level abundances of CDKs across models, while phosphorylation data is depicted in panel B. Panels C, D, and E display *ESR1* mutant enrichments for CDK activity (KSEA Z-score), CDK target (derived from KSEA output) protein enrichment (proteome), and phosphorylation status (phosphoproteome) for both estrogen stimulation and deprivation conditions, respectively. Panels F and G depict proteome and phosphoproteome levels of detected Cyclin proteins across cell models.

Acronyms: CDK: cyclin-dependent kinase; E2: 17-β-estradiol; ER: estrogen receptor; EtOH: ethanol; KSEA: kinase substrate enrichment analysis.


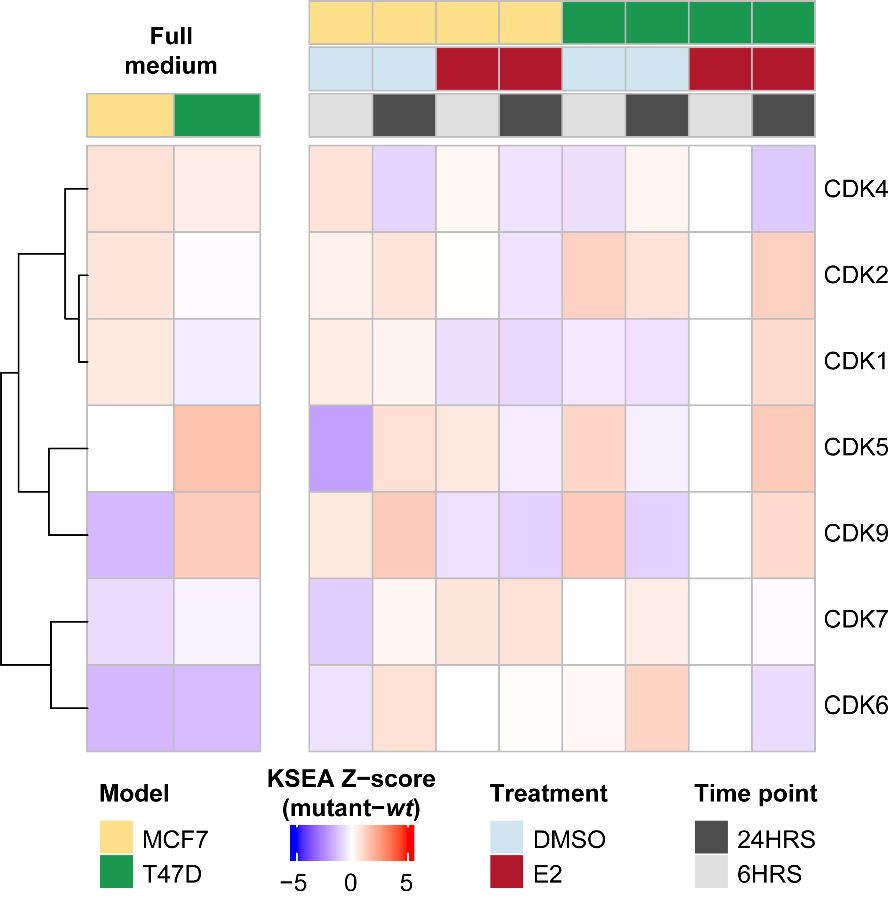


**Figure S10. CDK enrichment in the MCF7-T47D dataset.**

Heatmap displays KSEA Z-scores (mutant over *wt*) for CDKs out of our additional dataset (MCF7 and T47D models). Left heatmap depicts results for full medium data, while left heatmap displays data after estrogen deprivation or stimulation.

Acronyms: CDK: cyclin-dependent kinase; KSEA: kinase substrate enrichment analysis.


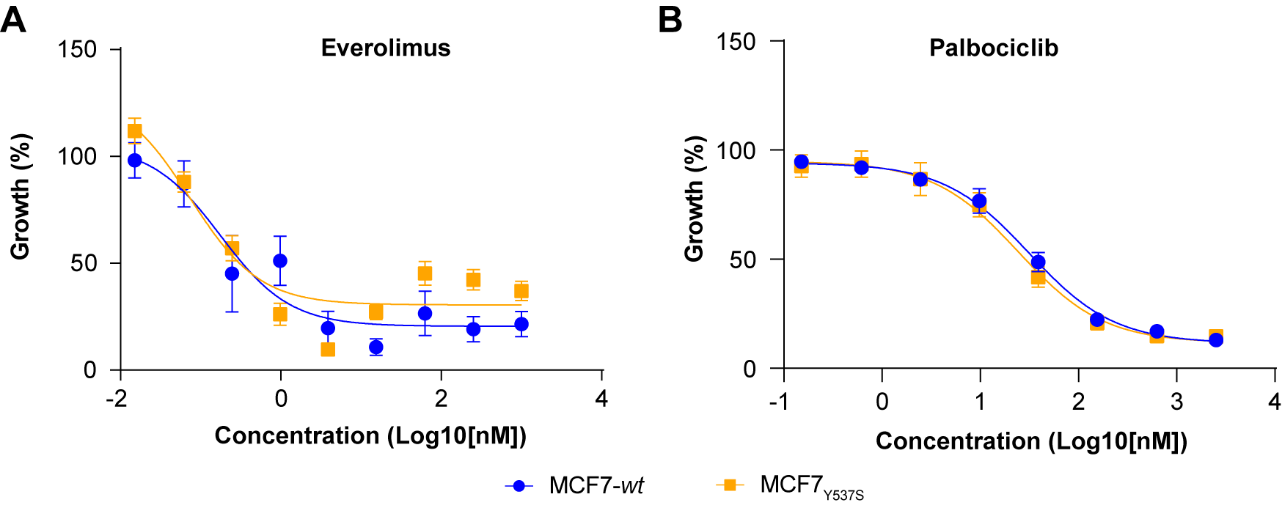


**Figure S11. Inhibition of mTOR and CDK pathways.**

Growth assays were performed for wt MCF7 and MCF7_Y537S_ cells in full media containing mTOR inhibitor Everolimus (panel A) and CDK4/6 inhibitor Palbociclib (panel B).


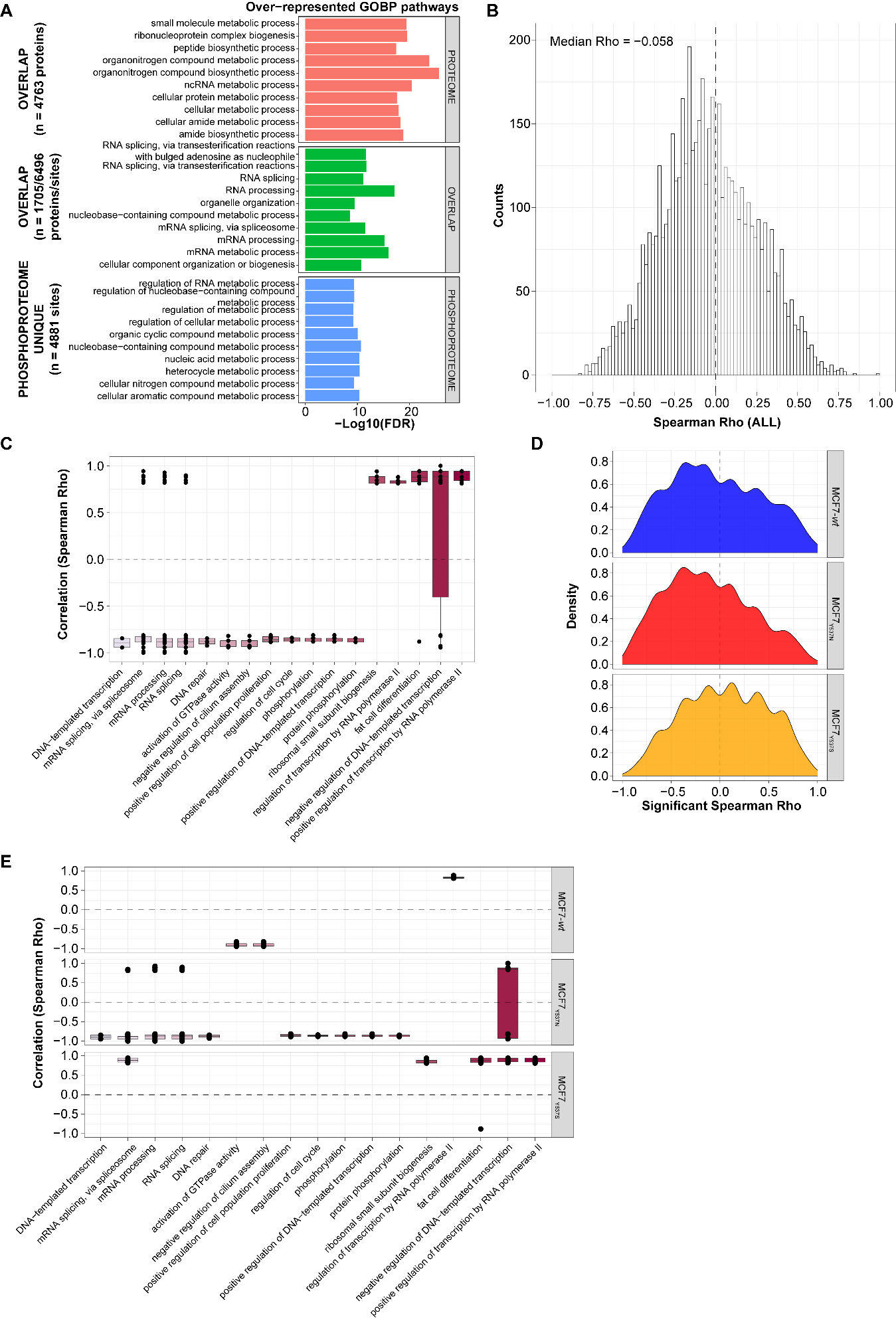


**Figure S12. Correlation analysis of proteome and phosphoproteome overlaps in our MCF7 dataset.**

Figure displays analysis of the overlap between the proteome and phosphoproteome datasets. Panel A displays the most (top 10) represented GOBP pathways in each subset. Panel B represents overall protein-phosphosite correlations across all samples, while panel C displays GOBP enrichment analysis for lowly and highly correlating protein-phosphosite pairs. Panel D displays protein-phosphosite correlation distributions across the MCF7 models, while panel E shows proteome-phosphoproteome correlation distributions (box-plots) of significantly enriched (correlation distribution test adjusted *p-value* < 0.05) GOBP pathways in each model.

Acronyms: GOBP: gene ontology biological process.

**
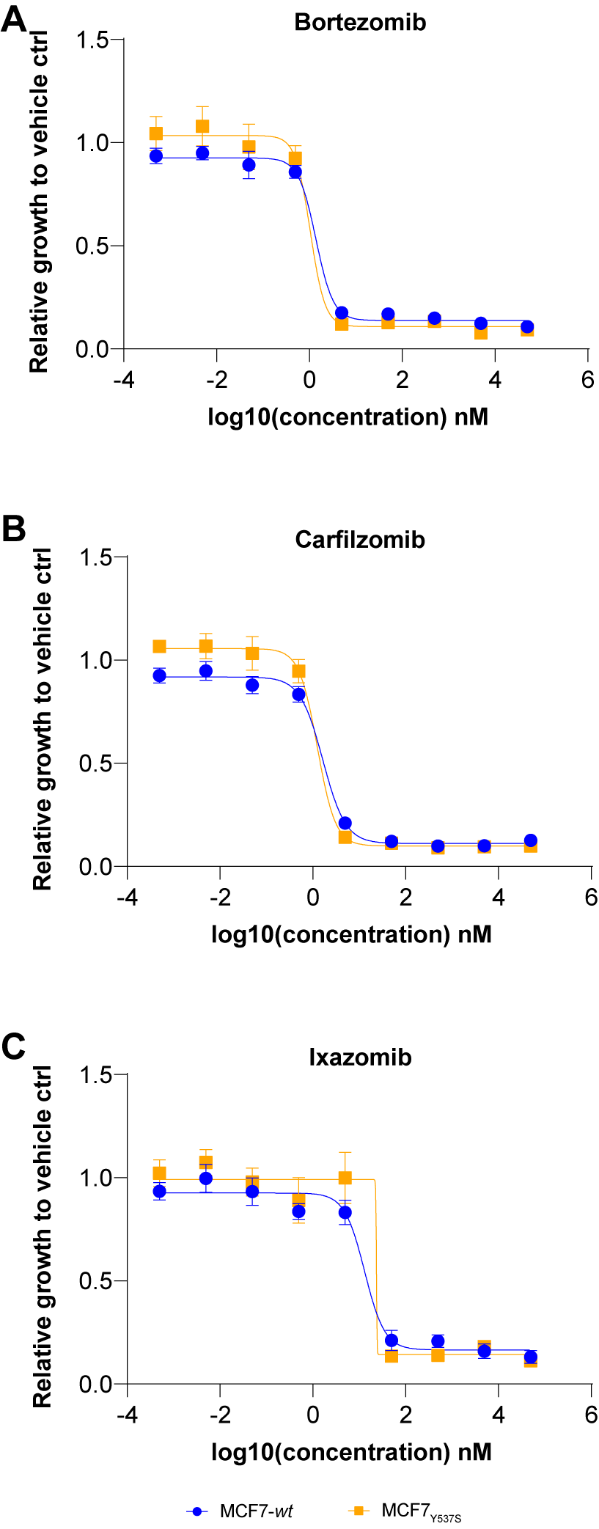
**

**Figure S13. Mono-therapy inhibition of the proteasome in MCF7 models.**

Growth assays were performed for *wt* MCF7 and MCF7_Y537S_ cells in full media containing proteasome inhibitors (panel A: Bortezomib; panel B: Carfilzomib; panel C: Ixazomib). Cell growth was measured by sulphorhodamine B assay.
